# The Human Developing Cerebral Cortex Is Characterized by an Elevated De Novo Expression of Long Noncoding RNAs in Excitatory Neurons

**DOI:** 10.1093/molbev/msae123

**Published:** 2024-06-24

**Authors:** David A Morales-Vicente, Ana C Tahira, Daisy Woellner-Santos, Murilo S Amaral, Maria G Berzoti-Coelho, Sergio Verjovski-Almeida

**Affiliations:** Laboratório de Ciclo Celular, Instituto Butantan, São Paulo, Brazil; Departamento de Bioquímica, Instituto de Química, Universidade de São Paulo, São Paulo, Brazil; Laboratório de Ciclo Celular, Instituto Butantan, São Paulo, Brazil; Laboratório de Ciclo Celular, Instituto Butantan, São Paulo, Brazil; Departamento de Bioquímica, Instituto de Química, Universidade de São Paulo, São Paulo, Brazil; Laboratório de Ciclo Celular, Instituto Butantan, São Paulo, Brazil; Laboratório de Ciclo Celular, Instituto Butantan, São Paulo, Brazil; Departamento de Bioquímica, Instituto de Química, Universidade de São Paulo, São Paulo, Brazil; Laboratório de Ciclo Celular, Instituto Butantan, São Paulo, Brazil; Departamento de Bioquímica, Instituto de Química, Universidade de São Paulo, São Paulo, Brazil

**Keywords:** cerebral cortex evolution, excitatory (glutamatergic) neurons, inhibitory (GABAergic) neurons, outer radial glial cells (oRGCs), lncRNA evolution

## Abstract

The outstanding human cognitive capacities are computed in the cerebral cortex, a mammalian-specific brain region and the place of massive biological innovation. Long noncoding RNAs have emerged as gene regulatory elements with higher evolutionary turnover than mRNAs. The many long noncoding RNAs identified in neural tissues make them candidates for molecular sources of cerebral cortex evolution and disease. Here, we characterized the genomic and cellular shifts that occurred during the evolution of the long noncoding RNA repertoire expressed in the developing cerebral cortex and explored putative roles for these long noncoding RNAs in the evolution of the human brain. Using transcriptomics and comparative genomics, we comprehensively annotated the cortical transcriptomes of humans, rhesus macaques, mice, and chickens and classified human cortical long noncoding RNAs into evolutionary groups as a function of their predicted minimal ages. Long noncoding RNA evolutionary groups showed differences in expression levels, splicing efficiencies, transposable element contents, genomic distributions, and transcription factor binding to their promoters. Furthermore, older long noncoding RNAs showed preferential expression in germinative zones, outer radial glial cells, and cortical inhibitory (GABAergic) neurons. In comparison, younger long noncoding RNAs showed preferential expression in cortical excitatory (glutamatergic) neurons, were enriched in primate and human-specific gene co-expression modules, and were dysregulated in neurodevelopmental disorders. These results suggest different evolutionary routes for older and younger cortical long noncoding RNAs, highlighting old long noncoding RNAs as a possible source of molecular evolution of conserved developmental programs; conversely, we propose that the de novo expression of primate- and human-specific young long noncoding RNAs is a putative source of molecular evolution and dysfunction of cortical excitatory neurons, warranting further investigation.

## Introduction

The cerebral cortex is a primary information-processing center of the central nervous system that is crucial to the evolution of higher cognition and is affected by neurodevelopmental disorders. It comprises billions of excitatory projection neurons (glutamatergic) and inhibitory interneurons (GABAergic) assembled in local circuits intertwined with glial and vascular cells arranged in a six-layered architecture on the outer surface of the mammalian brain (Molnár et al. 2019; Libé-Philippot and Vanderhaeghen 2021). The cerebral cortex evolved from the dorsal pallium after the divergence of mammals and sauropsids (reptiles and birds) approximately 300 million years ago (Mya), and it is endowed with incredible plasticity, evident in the diverse neocortical sizes and shapes (Lui et al. 2011; Silbereis John et al. 2016). Primates present an expanded brain with more total neurons than most mammalian species. The human cerebral cortex has further expanded, differentiating us from our closest living relatives. These expansions and the diversification of neuronal cell types are likely responsible for the computational capacities and unparalleled cognition of humans (Berg et al. 2021).

At the cellular level, the human developing cerebral cortex presents an augmented proliferative capacity of neural progenitors (radial glial cells [RGCs]), especially from the outer subventricular zone (outer RGCs [oRGCs]; Lui et al. 2011), as well as an improvement in the information processing capability of mature excitatory neurons (Berg et al. 2021). Understanding the molecular basis of these differences is critical to unveiling the evolution of human higher cognition and having a deeper comprehension of how they are disrupted in diseases (Silbereis John et al. 2016). In this line, a considerable effort has been made in the past decade to identify those changes, finding that duplications of protein-coding genes and modifications in gene regulatory regions have altered the transcriptional landscape of different cell populations in the developing cerebral cortex (Libé-Philippot and Vanderhaeghen 2021; Vanderhaeghen and Polleux 2023). This extensive work has mainly focused on changes in the expression of protein-coding genes; expanding this analysis to the human noncoding transcriptome is crucial to improve our understanding of the gene regulatory modifications that have led to the evolution of the human cerebral cortex.

Long noncoding RNAs (lncRNAs) are noncoding genes transcribed into RNAs longer than 200 nucleotides that do not translate into functional proteins. This heterogeneous group of RNAs is transcribed by RNA pol II and shares molecular features with mRNAs, such as being 5′ capped, spliced, and polyadenylated; despite the molecular similarities with mRNAs, lncRNAs also present features that differentiate them, including higher tissue specificity, distinct chromatin modifications at the promoter region, cell nucleus enrichment, inefficient splicing, and less stability compared with mRNAs (Ransohoff et al. 2018; Statello et al. 2021). Although these features may point to lncRNAs as mere transcriptional noise, it has been shown that at least a fraction of lncRNAs and the act of their transcription have gene regulatory functions (Rinn and Chang 2020). Interestingly, neural tissues express a significant number of lncRNAs in tetrapods (Necsulea et al. 2014; Hezroni et al. 2015; Sarropoulos et al. 2019), and several lncRNAs have been characterized as functional regulatory RNAs of different stages of the cerebral cortex development (Aprea and Calegari 2015).

Unlike protein-coding genes that have evolved mainly by gene duplications, lncRNAs have preferentially evolved by de novo expression and exonization mediated by transposable elements (TEs; Kapusta et al. 2013). The de novo expression and the significant contributions of TEs to the evolution of lncRNAs explain the reduced constraint under which lncRNAs evolved compared with protein-coding genes (Kapusta et al. 2013). In addition, it has been shown in mammals that lncRNAs are a source of cellular plasticity due to their capacity to acquire new functional modalities (Guo et al. 2020), and some lncRNAs are a source of newly identified peptides (Ruiz-Orera et al. 2014). Interestingly, the first highly evolving human-specific region (human accelerated region [HAR]) was identified inside the lncRNA *HAR1F*, expressed in the developing cerebral cortex (Pollard et al. 2006). This faster evolutionary turnover of lncRNAs compared with mRNAs, their gene regulatory functions, and elevated expression in neural tissues make lncRNAs good candidates for molecular drivers of biological innovations in the context of cerebral cortex evolution.

Here, we used transcriptomics and comparative genomics to characterize the evolution of the lncRNA repertoire of the developing cerebral cortex. Our analyses identified signatures of different evolutionary routes by which lncRNAs were born throughout human evolution. They suggested that old and young lncRNAs are possible sources of molecular innovation of conserved and divergent developmental programs in the cerebral cortex, respectively.

## Results

### Assemblies of New Comprehensive Transcriptomes Improve the Annotation of lncRNA Genes in Humans and Other Three Vertebrate Model Organisms

To properly characterize the evolution of human cortical lncRNAs, we set out to compare the human lncRNA repertory to those of three other vertebrate species (rhesus macaque, mouse, and chicken) that helps to recapitulate the evolutionary history of the human cerebral cortex. The vertebrate nervous system contains more specific lncRNAs than most other body tissues (Necsulea et al. 2014; Hezroni et al. 2015; Sarropoulos et al. 2019). However, the lack of extensive RNA-seq libraries from the prenatal cerebral cortex has hampered the reconstruction of more representative lncRNA gene models for this brain region. lncRNAs are more tissue specific and expressed at lower levels than mRNAs, and many cell types (particularly those that are rare or found in early embryonic stages) have not yet been thoroughly interrogated by RNA-seq (Ulitsky 2016). Consequently, to avoid misidentifying homologous lncRNAs due to differences in the completeness of the human, rhesus macaque, mouse, and chicken transcriptome annotations, we generated and annotated new comprehensive catalogs of lncRNAs for all four species.

We gathered extensive RNA-seq libraries from healthy tissues encompassing different stages of cerebral cortex and cerebral pallium development. This collection of RNA-seq libraries includes recently published data sets from humans, rhesus macaques, and mice (Li et al. 2018; Zhu et al. 2018; Sarropoulos et al. 2019; supplementary fig. S1a to c and table S1, Supplementary Material online); additionally, we generated new bulk RNA-seq data from the chicken pallium (supplementary fig. S1d and table S1, Supplementary Material online). We developed a genome-assisted approach that integrates efficient and more accurate bioinformatics tools that we applied to the collected short-read RNA-seq libraries (see Materials and Methods and supplementary fig. S2a, Supplementary Material online). In brief, we mapped bulk RNA-seq short reads to the genome with *STAR* (Dobin et al. 2013), using the latest available genomes as references (hg38, rheMac10 [Warren et al. 2020], mm39 [https://www.ncbi.nlm.nih.gov/grc/mouse], and galGal6 [Bellott et al. 2017]), most of which were based on long-read sequencing. We removed RNA-seq libraries with high 3′ bias (see Materials and Methods and supplementary figs. S2a and S3a to d, Supplementary Material online), and filtered libraries were used to assemble new transcriptome models for all species. Then, we generated transcript models from each library using *Scallop* (Shao and Kingsford 2017). To avoid annotating transcriptional artifacts, we removed all unspliced transcripts. New consensus transcript models from all libraries for each developmental stage/brain region (supplementary fig. S1, Supplementary Material online) were generated using *TACO* (Niknafs et al. 2017). A final short-read–based consensus set of transcript models was again reconstructed from the previous developmental stage/brain region models using *TACO*. To help improve the transcriptome assembly for rhesus macaque and chicken based on short reads, we collected publicly available IsoSeq long-read libraries (see Materials and Methods and supplementary fig. S3b, Supplementary Material online), mapped them to the corresponding genomes using *Minimap2* (Li 2018), and generated transcriptome models using *Cupcake* (https://github.com/Magdoll/cDNA_Cupcake/). A final consensus set of transcript models was obtained by merging the short-read, long-read–based, and *Ensembl/Gencode* (Frankish et al. 2019; Yates et al. 2019) transcript models using *gffcompare* (Pertea and Pertea 2020).

Subsequently, we applied a stringent criterion in all four species to identify protein-coding potential in each transcript (see Material and Methods and supplementary fig. S2, Supplementary Material online). In brief, we used three different coding potential calculators: *CPC2*, *CPAT3*, and *Feelnc* (Wang et al. 2013; Kang et al. 2017; Wucher et al. 2017). Additionally, we identified bona fide open reading frames (ORFs) using *Transdecoder* (Douglas 2018), *blast* (Camacho et al. 2009), and *Pfam* (El-Gebali et al. 2019; see Materials and Methods and supplementary fig. S3c, Supplementary Material online). Finally, to annotate newly identified ORFs, we used *eggNOG-mapper* (Huerta-Cepas et al. 2017). Transcripts were identified as lncRNAs if up to one of the three coding potential calculators classified the transcript as protein coding, and there was no bona fide ORF identified with *Transdecoder*. A locus was annotated as lncRNA if all transcripts from the same locus were classified as lncRNAs or if the Gencode annotation for human and mouse transcriptomes already annotated the locus as containing a lncRNA gene (detailed coding potential information for each transcript can be found at https://doi.org/10.5281/zenodo.10038370). To generate the final comprehensive catalog of lncRNAs of the four species, lncRNAs from public lncRNA databanks (Hezroni et al. 2015; Sarropoulos et al. 2019) were incorporated into the final transcriptomes using *gffcompare*. The gtf files of the new transcriptomes are available at https://doi.org/10.5281/zenodo.10038370.

Of these new transcriptomes, lncRNAs represent the category with more annotated genes (supplementary fig. S4a to d, Supplementary Material online), corroborating the widespread expression of lncRNAs in vertebrates (Necsulea et al. 2014; Hezroni et al. 2015; Sarropoulos et al. 2019). In our human transcriptome assembly, a final set of 108,386 genes was identified and annotated, of which 65,639 were annotated as lncRNAs mapped to the primary chromosomes (supplementary table S2, Supplementary Material online). Of note, our new assemblies annotated 27,966 new lncRNA loci (42% of 65,639 lncRNAs in the assembly) in humans, 17,240 new lncRNAs (40.4% of 42,684 lncRNAs) in rhesus macaques, 11,967 new lncRNAs (25% of 45,573 lncRNAs) in mice, and 13,245 new lncRNAs (53.4% of 24,796 lncRNAs) in chickens (supplementary fig. S4e to h, Supplementary Material online and tables S2 and S3, Supplementary Material online) compared with Ensembl/Gencode, Hezroni et al. (2015), and Sarropoulos et al. (2019) data banks.

In addition, we used the *gffcompare* tool to compare the lncRNAs in our assemblies with the lncRNAs annotated in RNAcentral (RNAcentral_Consortium 2021), a curated lncRNAs database. Our assembly contained 28,334 lncRNAs (43% of 65,639 lncRNAs in the human assembly) not present in the RNAcentral human data set (supplementary fig. S5 and table S2, Supplementary Material online), 38,312 lncRNAs (90% of 42,684 lncRNAs in the macaque assembly) not present in the RNAcentral rhesus macaque data set, 34,761 lncRNAs (76% of 45,573 lncRNAs) not present in the mouse data set, and 24,785 lncRNAs (99.9% of 24,796 lncRNAs) not present in the chicken data set (supplementary fig. S5 and table S3, Supplementary Material online). Remarkably, we significantly improved the number of reads mapped to annotated features (supplementary fig. S4i to l, Supplementary material online). In addition to contributing to the annotation of new lncRNAs, we identified new protein-coding genes, pseudogenes, and other gene types, showing the robustness of our approach for identifying new coding and noncoding transcripts across different organisms, especially for species with poorly annotated transcriptomes, such as chicken and rhesus macaque.

### Sequence and Positional Conservation of lncRNA Genes Allows the Classification of Human Cortical lncRNAs into MA Groups

**Fig. 1.**
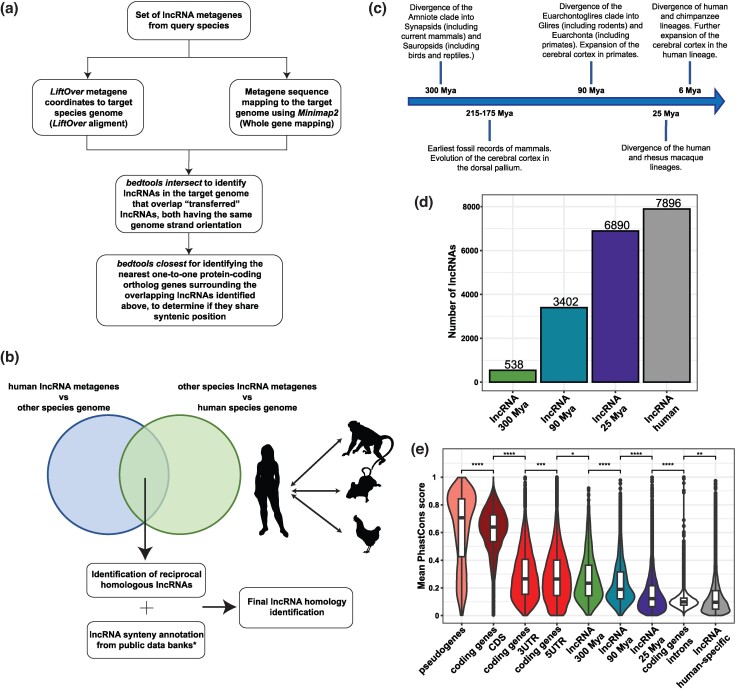
Classification of cortical lncRNAs into MA groups. a) Depiction of the two approaches used to identify homologous lncRNAs between two species: *LiftOver* alignment and whole-gene sequence mapping. b) Workflow for the identification of reciprocal homologous lncRNAs. (*) LncRNAs syntenic annotation from public data banks is specified in the Methods. c) Landmarks of cerebral cortex evolution in the human lineage. d) Distribution of the lncRNA genes in each MA group category. e) Mean PhastCons conservation scores of protein-coding genes, pseudogenes, and lncRNA MA groups. Statistics: All statistics are one-sided (greater) Wilcoxon tests. ns: *P* > 0.05; *P ≤ 0.05; **P ≤ 0.01; ***P ≤ 0.001; ****P ≤ 0.0001.

The fast evolutionary turnover of lncRNAs at a primary sequence level precludes the identification of orthologs in other species using conventional approaches, particularly for species that have diverged for long evolutionary periods. Syntenic conservation of lncRNAs between two species (conserved genomic position) has been used in the literature to improve the identification of homologous lncRNAs, allowing the discovery of conserved lncRNAs across vertebrates (Ulitsky et al. 2011; Hezroni et al. 2015). Inspired by these syntenic approaches, we developed a bioinformatic pipeline to systematically annotate lncRNAs into minimal evolutionary age (MA) groups based on genomic positional and sequence conservation of human lncRNA genes in the rhesus macaque, mouse, and chicken genomes (Fig. 1a and b; see Material and Methods). To reduce the chance of type I errors, we required that the set of positional homologous lncRNAs identified when using the human lncRNA transcriptome as a query be the same when using the other species as a query, thus identifying homologous lncRNAs only when having reciprocal positional conservation signals (Fig. 1b). To complement our homologs identification, we incorporated syntenic conservation classifications from lncRNAs in public databases (Ulitsky et al. 2011; Hezroni et al. 2015; Bryzghalov et al. 2020) into our data set (Fig. 1b). For each lncRNA gene, the oldest homology classification was retained if our classification differed (supplementary table S2, Supplementary Material online).

The human lncRNAs identified as homologous to chicken, mouse, and macaque lncRNAs were clustered into 300, 90, and 25 Mya MA groups, respectively (supplementary table S2, Supplementary Material online). lncRNAs that did not share conservation with any of the three above species or were not annotated as syntenic conserved with any species in public databases were classified as human-specific lncRNAs (supplementary table S2, Supplementary Material online). Other lncRNAs were classified as having uncertain evolutionary age and not further considered for subsequent analysis if they presented nonreciprocal conservation and were not annotated as syntenic conserved in public databases (supplementary table S2, Supplementary Material online). The MA groups were clustered around landmarks of cerebral cortex evolution: before and after the evolution of the cerebral cortex from the dorsal pallium, throughout the expansion of the cerebral cortex in the primate lineage, and during the current evolution of the cerebral cortex in humans (Fig. 1c).

After classifying the human lncRNA repertoire into MA groups, we identified the set of genes expressed throughout the development of the cerebral cortex using a total of 189 bulk RNA-seq libraries from samples of prenatal specimens from the PsychEncode project (Li et al. 2018) and two public single-cell RNA-seq data sets from the developing cerebral cortex (Fan et al. 2018; Zhong et al. 2018). Genes were identified as cortical genes if they were expressed with at least 0.5 transcripts per million (TPM) (supplementary table S2, Supplementary Material online) in all samples considered at each developmental window/region pair (Table 1) or if they were detected as differentially expressed (DE) in one single-cell cluster (supplementary table S4, Supplementary Material online). A final number of 40,348 genes were detected as expressed in the developing cerebral cortex (supplementary table S2, Supplementary Material online): 20,544 lncRNAs, 16,056 protein-coding genes, 3,259 pseudogenes, 482 genes of unknown coding potential, and seven genes annotated as “other” gene type. Out of the total 20,544 cortical lncRNAs, 18,726 were classified into one of four MA groups (Fig. 1d and supplementary table S2, Supplementary Material online); of the remaining 1,818 cortical lncRNAs, we classified 194 as being homologous to Apes and 1,624 as of uncertain evolutionary age.

**Table 1:** Human cerebral cortex developmental window-region pairs

Original brain region	PsychEncode developmental window				
	W1^a^	W2^a^	W3^a^	W4^a^	W5^a^
Dorsolateral prefrontal cortex	2	6	4	4	3
Medial prefrontal cortex	2	6	4	4	4
Orbital prefrontal cortex	2	6	2	3	4
Ventrolateral prefrontal cortex	0	6	4	4	4
Primary motor-somatosensory cortex	2	0	3	0	0
Primary motor (m1) cortex	0	6	1	3	4
Primary somatosensory (s1) cortex	0	6	0	3	4
Occipital neocortex	2	0	0	0	0
Primary visual (v1) cortex	0	6	4	3	4
Parietal cortex	1	0	0	0	0
Posterior inferior parietal cortex	0	6	4	3	4
Primary auditory (a1) cortex	0	6	4	3	4
Inferior temporal cortex	0	6	2	2	4
Superior temporal cortex	0	4	4	3	4

^a^Number of RNA-seq libraries that were used per brain region (indicated at left) at each developmental window (Wn, indicated at top) for identifying the expression of lncRNAs in the developing cerebral cortex, from a total of 189 libraries (see supplementary table S1, Supplementary Material online).

The number of cortical lncRNAs classified into the four MA groups increases throughout the evolution of the human lineage, with only 2.6% of them identified as appearing before the evolution of the cerebral cortex and approximately 43% of them being specific to humans (Fig. 1d), in line with the fast evolutionary turnover described for lncRNAs (Necsulea et al. 2014; Hezroni et al. 2015).

**Fig. 2.**
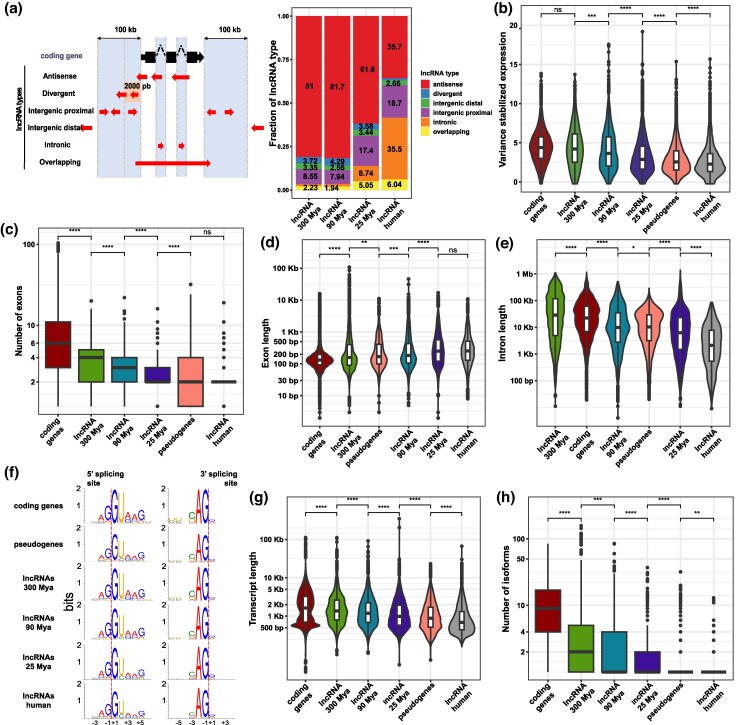
Differences in the genomic features of cortical lncRNAs. a) Left, schematic depiction of the classification of lncRNAs based on their position regarding protein-coding genes; right, distribution of lncRNA types among the lncRNA MA groups. b) Expression levels of protein-coding genes, pseudogenes, and lncRNA MA groups. c) Number of exons distribution among protein-coding genes, pseudogenes, and lncRNA MA groups. d to e) Like c) but showing the exon and intron length distribution, respectively. f) Frequency of splicing motifs among protein-coding genes, pseudogenes, and lncRNA MA groups. g to h) Transcript length distribution and the number of isoforms among protein-coding genes, pseudogenes, and lncRNA MA groups. Statistics: All statistics are one-sided (greater) Wilcoxon tests. ns: *P* > 0.05; *P ≤ 0.05; **P ≤ 0.01; ***P ≤ 0.001; ****P ≤ 0.0001.

Next, we checked the phastCons scores to test the conservation status among MA groups, finding that these scores significantly decreased throughout the evolution of cortical lncRNAs (Fig. 1e). lncRNA genes were further classified depending on their position in the genome relative to protein-coding genes (Fig. 2a, left). The relative abundance of lncRNA types changed through evolution, with older lncRNAs being mostly antisense; meanwhile, overlapping, intergenic proximal, and intronic lncRNA fractions increased in the younger lncRNAs (Fig. 2a, right). Significantly, the number of intronic lncRNAs has increased in the human lineage (Fig. 2a, right), as 80.9% of the total intronic lncRNAs are human-specific.

### Older lncRNAs Have Enhanced Expression Strength, Splicing Efficiency, and Loci Complexity

After classifying the cortical lncRNAs into the MA groups, we assessed the differences in their genomic features. We found that the MA groups follow an expression gradient, where older lncRNAs reach more robust expression levels in the human cortex than younger lncRNAs (Fig. 2b). Interestingly, the oldest group of cortical lncRNAs achieves similar expression levels to protein-coding genes (Fig. 2b), which shows that the general lower expression of lncRNAs compared with protein-coding genes (Necsulea et al. 2014; Hezroni et al. 2015) is masked by the significant difference in gene expression levels between old and young lncRNAs.

There are documented differences in the literature between mRNA and lncRNA splicing efficiency (Movassat et al. 2019). Therefore, we decided to assess differences in splicing efficiency between mRNAs and lncRNAs among the MA groups by evaluating the number of exons, exon lengths, and intron lengths. To reduce the possibility of a confounding effect of expression level and lncRNA type in this analysis, we evaluated only a set of expression- and type-matched genes within a given MA group (supplementary fig. S6a and b, Supplementary Material online). Like the gene expression levels, the average number of exons of a lncRNA among MA groups follows a gradient, where older lncRNA populations have significantly more exons on average than younger populations (Fig. 2c). Nevertheless, in contrast to expression levels, mRNAs present a considerably higher number of exons than all lncRNA MA groups (Fig. 2c).

Exon and intron lengths were also investigated, and we found that older lncRNAs have significantly shorter exon lengths and longer intron lengths than younger lncRNAs (Fig. 2d and e), while mRNAs have, in general, shorter exon lengths than all lncRNA MA groups (Fig. 2d); interestingly, the oldest MA group has longer introns than mRNAs (Fig. 2e). It has been described that in humans, last exons are on average longer than first exons and that internal exons have on average the shortest lengths (Movassat et al. 2019). We have observed that the pattern of shorter exon lengths of older lncRNAs and mRNAs holds only for the first exons and internal exons (supplementary fig. S6c, Supplementary Material online), while for the last exons, the pattern is inverted, and the younger lncRNAs have the shortest exons (supplementary fig. S6c, Supplementary Material online). These differences in exon and intron lengths among MA groups suggest differences in their splicing efficiency, so we assessed the frequency of canonical splicing motifs in the lncRNA groups. We found that older lncRNAs present a higher frequency of stronger splicing motifs than younger lncRNAs, especially affecting the 5′ splice sites (Fig. 2f). In summary, these features suggest that canonical splicing motifs (Sheth et al. 2006; Parada et al. 2014) are more efficiently processed in older lncRNAs.

It has been shown that longer transcripts have features of dynamic expression associated with lncRNA functionality (Hezroni et al. 2015; Sarropoulos et al. 2019). We assessed the length of the transcripts and the number of isoforms among the MA groups; older lncRNAs have significantly longer transcripts and significantly more isoforms than younger lncRNAs (Fig. 2g and h), indicating an increase in locus complexity for older lncRNAs; however, all lncRNAs have reduced locus complexity compared with protein-coding genes (Fig. 2h).

### lncRNA Evolutionary Groups Show Distinct Distributions of TE Insertions But Shared Nuclear Retention

**Fig. 3.**
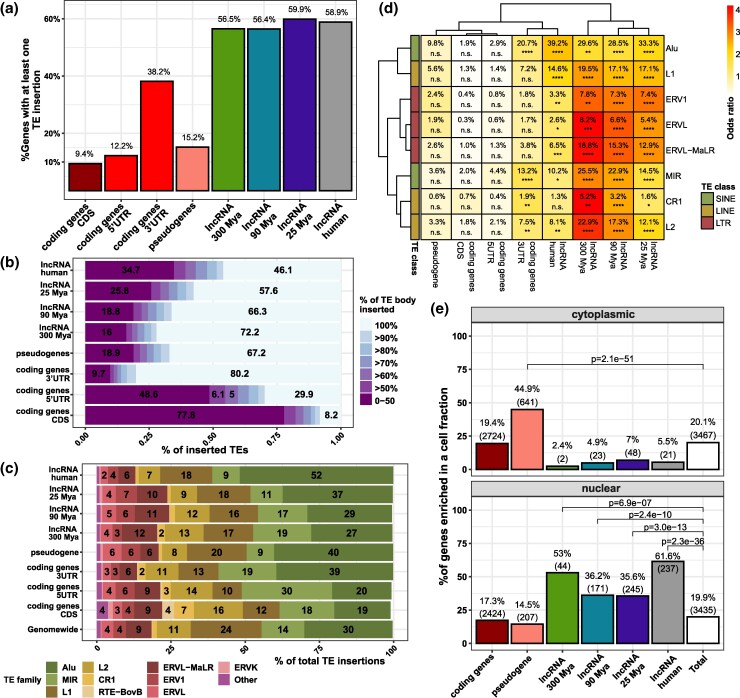
TE insertion features of cortical lncRNAs throughout evolution. a) Percentage of genes carrying at least one TE insertion. b) For each gene type indicated at left, the horizontal bars show the fraction of inserted TEs with the indicated % of TE body inserted into the gene body (colored according to the scale at right). c) Percentage of each TE family (color coded as shown at the bottom) inserted into each gene type indicated at left. d) Heatmap displaying the percentage of genes in a category carrying at least one TE family insertion. Heatmap colors are proportional to the odds ratio statistics of enrichment of each TE family in a category. e) Percentage of genes DE in the cellular compartment indicated at the top of each panel. *P*-values are Fisher's hypergeometric test. Statistics: All statistics labels are FDR-corrected *P*-values of one-sided (greater) Fisher hypergeometric tests. *P ≤ 0.05; **P ≤ 10^−5^; ***P ≤ 10^−10^; ****P ≤ 10^−15^.

Transposable elements (TEs) are the main drivers of lncRNA diversification and evolution (Kapusta et al. 2013; Hezroni et al. 2015); therefore, TEs might be involved in the differences in transcript complexity among lncRNA MA groups. To evaluate this scenario, the incidence of TE insertions was assessed. Interestingly, all lncRNAs show a similar percentage of TE occurrence (56.5% to 58.9%; Fig. 3a) that differs from the depletion of TE insertion in pseudogenes and protein-coding genes; nevertheless, 3′ untranslated regions (UTRs) present an increased incidence of TE insertions (Fig. 3a). The extent of the TE body inserted in genes differs among lncRNA MA groups; older lncRNAs incorporated larger fractions (72.2%) of full-length TE sequences than younger lncRNAs (46.1% to 66.3%; Fig. 3b).

It has been hypothesized that TE-derived exons have evolved as RNA domains essential for lncRNA function (Johnson and Guigó 2014). We assessed the distribution of TE families among the different gene categories; all gene categories showed deviations from the overall genome-wide distribution of TE families (Fig. 3c). The LINE family L1, the second most abundant TE in the genome, was reduced from all considered gene types (Fig. 3c), especially from protein-coding genes; interestingly, pseudogenes are genes with the highest frequency of L1 insertion (Fig. 3c), pointing to L1 insertion as a mark of pseudogenization of coding genes. Moreover, most of the lncRNA categories, except the human-specific lncRNAs, showed a markedly higher frequency of endogenous retroviruses (ERVs), particularly the ERVL-MaLR family, which is the most abundant ERV in the human genome (Fig. 3c). Furthermore, we evaluated the percentage of genes carrying at least one TE family insertion among gene categories and identified that ERVs are more often present in the gene body of all cortical lncRNAs (Fig. 3d).

Remarkably, L1 and Alu, active TEs of the human genome (Mills et al. 2007), are the TE families more frequently present in human-specific lncRNAs (Fig. 3c and d); in particular, Alu represents more than half of the TE insertion of this group of lncRNAs, while inactive TE families are enriched in older lncRNAs (Fig. 3c and d). This trend of TE family enrichment follows a gradient, where Alu occurrence among lncRNA MA groups is more prevalent in younger lncRNAs; inversely, inactive TE families such as ERVs, L2, CR1, and MIR are more prevalent in older lncRNAs than in younger lncRNAs (Fig. 3c and d).

Several TE families have been recognized as signals of nuclear retention of lncRNAs (Lubelsky and Ulitsky 2018; Carlevaro-Fita et al. 2019). Due to the differences in the distribution of TE families among the lncRNA MA groups, the nuclear retention feature of lncRNAs might also differ. Thus, we assessed the distribution of lncRNAs among the nuclear and cytoplasmic compartments in human fetal cortical tissues (Fig. 3e and supplementary tables S1 and S5, Supplementary Material online). Remarkably, lncRNAs of all MA groups were proportionally more expressed in the nucleus than in the cytoplasm (Fig. 3e); regarding the lncRNAs expressed in the nucleus, different levels of expression resulted in lncRNA fractions varying from 35.6% to 61.6% among the different lncRNA MA groups. These results show that nuclear retention is a feature that has prevailed in lncRNAs through evolution.

### lncRNA Evolutionary Groups Show Distinct Genomic Distributions That Suggest a Potential Functional Specialization

**Fig. 4.**
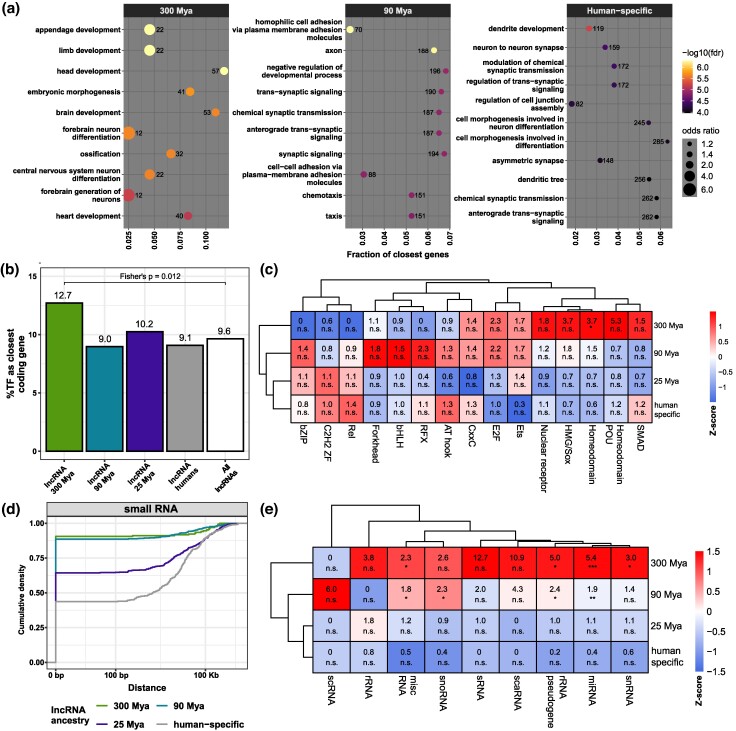
Differential genomic distribution of cortical lncRNAs. a) Top 10 GO terms enriched in the sets of protein-coding genes closest to the different lncRNA MA groups indicated at the top of each panel. The number of closest protein-coding genes identified in each GO category is given next to the circle; the significance of the enrichment is given by the color of the circle, according to the color scale at the right of the panel. b) Percentage of TFs as the closest genes for each lncRNA MA group. c) Percentage of TF as the nearest gene separated by TF families, indicated in the columns; the displayed number indicates the odds ratio across of MA groups. Heatmap colors are proportional to the scaled odds ratio across TF family. d) Cumulative distribution of the distance from the lncRNA to the nearest small RNA separated by MA groups. e) Like c) but for small RNAs as the closest gene. Statistics: All statistics labels are FDR-corrected *P*-values of one-sided (greater) Fisher hypergeometric tests. ns: *P* > 0.05; *P ≤ 0.05; **P ≤ 10^−5^; ***P ≤ 10^−10^. miRNA, microRNA; rRNA, ribosomal RNA; scaRNA, small Cajal body-specific RNA; scRNA, small cytoplasmic RNA; snoRNA, small nucleolar RNA; snRNA, small nuclear RNA; sRNA, small RNA.

The local genomic context of lncRNAs is intimately associated with their functionality (Engreitz et al. 2016; Amaral et al. 2018). Thus, systematically inspecting protein-coding genes proximal to lncRNAs of different MA groups might shed light on the function cortical lncRNAs might have gained throughout evolution. For that, the nearest protein-coding genes were retrieved to a hundred kilobases surrounding the lncRNAs of different MA groups; then, we assessed their enriched gene ontology (GO) terms. We found that MA groups evolved from loci near distinct types of developmental protein-coding genes that regulate different stages of neuron specification and maturation (Fig. 4a and supplementary table S6, Supplementary Material online). Thus, old lncRNAs that appeared before the evolution of the cerebral cortex (300 Mya) tend to occupy loci near developmental regulatory genes, including transcription factors (TFs) (Fig. 4a, left; supplementary table S6, Supplementary Material online); meanwhile, a fraction of lncRNAs that appeared before the expansion of the primate cerebral cortex (90 Mya) are expressed from loci near protein-coding genes associated with the development of axons (Fig. 4a, center; supplementary table S6, Supplementary Material online), and a fraction of younger human-specific lncRNAs are expressed from loci proximal to genes associated with dendrite development (Fig. 4a, right; supplementary table S6, Supplementary Material online), where synapses are finely tuned in response to environmental cues (Citri and Malenka 2008).

Furthermore, one of the enriched GO terms of protein-coding genes from the vicinity of old cortical lncRNAs was “DNA-binding transcription activator activity” (supplementary table S6, Supplementary Material online); therefore, we tested whether older lncRNAs have an enriched number of TF loci in their proximity, finding that only the older MA group has a slightly increased abundance of proximal TF loci compared with the other MA groups (Fig. 4b). Due to the considerable fraction of TFs as the closest coding gene (9.0% to 12.7%) and the fact that TFs are the master regulators of biological functioning, we sought to identify the type of TFs proximal to lncRNAs of different MA groups. Surprisingly, lncRNAs of different MA groups are preferentially distributed around certain families of TFs. Many older lncRNAs are expressed from loci close to the homeodomain-containing TFs (Fig. 4c), master regulators of early development. Meanwhile, younger lncRNAs are expressed from loci near C2H2 zinc finger-containing TFs, a fast-evolving family of transcriptional repressors (Tadepally et al. 2008; Najafabadi et al. 2015;  Fig. 4c).

We observed that older lncRNAs (300 and 90 Mya) are more often proximal to small RNAs than younger lncRNAs (25 Mya and human-specific; Fig. 4d); it has been shown that several lncRNAs host small RNAs (Sun et al. 2021). Therefore, we tested whether older lncRNAs were sources of small RNA genes and found that older lncRNAs preferentially host small RNAs in their loci (Fig. 4e), especially microRNAs, which suggests a possible coevolution of older cortical lncRNAs with microRNAs.

### lncRNA Evolutionary Groups Exhibit Different Expression Patterns and Cellular Enrichment

**Fig. 5.**
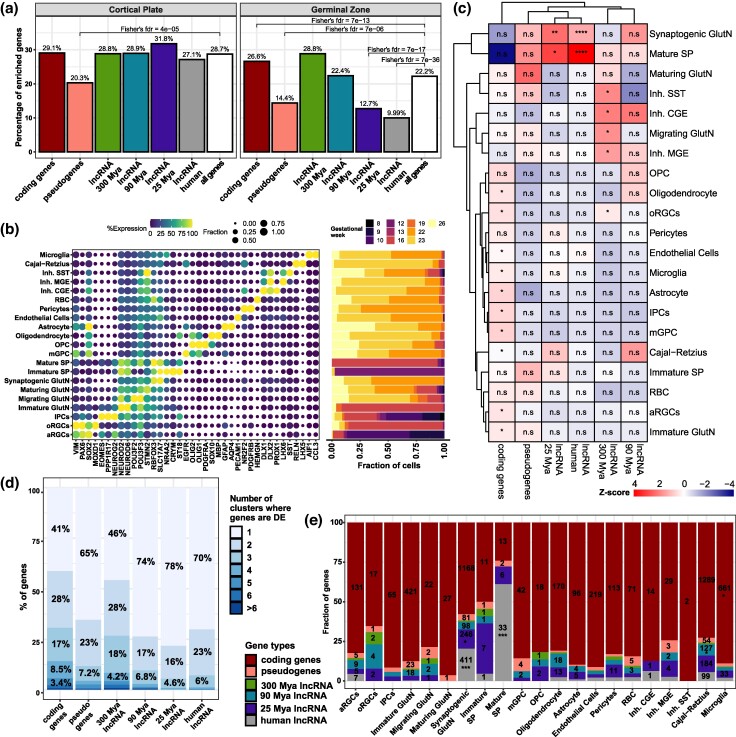
Differential distribution of lncRNAs in cortical cell types throughout evolution. a) Percentage of DE enriched genes by gene category in cortical plate (left) or germinal (right) zones of mid-gestation cerebral cortices. FDR values on the enriched gene categories come from Fisher's hypergeometric test. b) Dotplot (left) showing the average expression of the cell population marker genes (*x* axis) related to the single-cell cluster (*y* axis) with the maximum expression and barplot (right) displaying the fraction of cells from a cluster coming from a gestational week (right). c) Heatmap displaying the MA groups (columns) identified in our analyses as enriched in each cell type (indicated on the right). Heatmap colors are proportional to the *z*-score across MA groups of the percentage of the distinct gene types in each cell type. d) Frequency of the cell-type specificity of different gene types; for each gene type (column), the percentage of those genes that are DE in 1 to 6 or more cell clusters (as indicated by the color) is given inside the boxes; these DEGs are specific markers of those cell clusters. e) Frequency of cell-type–specific genes colored by gene type (color scale at left). Cell types are indicated at the *x* axis; numbers inside the bars indicate the absolute number of cell-type–specific lncRNAs in each cell type cluster. Statistics: All statistics labels are FDR-corrected *P*-values of one-sided (greater) Fisher hypergeometric tests. ns: *P* > 0.05; *P ≤ 0.05; **P ≤ 10^−5^; ***P ≤ 10^−10^; ****P ≤ 10^−15^. aRGCs, apical radial glial cells; GlutN, glutamatergic neurons; Inh. CGE, inhibitory GABAergic interneurons derived from the caudal ganglionic eminences; Inh. MGE, inhibitory GABAergic interneurons derived from the medial ganglionic eminences; Inh. SST, inhibitory GABAergic interneurons expressing somatostatin; IPCs, intermediate progenitor cells; mGPC, multipotent glial progenitor cells; OPC, oligodendrocyte progenitor cells; oRGCs, outer radial glial cells; RBC, red blood cells; SP, subplate.

We have shown that cortical lncRNAs display commonalities, differentiating them from mRNAs and pseudogenes. They also show differences among MA groups (locus complexity, TE composition, and genomic distribution) that point to differences in their evolutionary history. However, the extent to which lncRNA evolution might have impacted the biology of the cerebral cortex remains unexplored. To address this, we first assessed the expression pattern of lncRNAs from the different MA groups in a set of mid-gestational RNA-seq libraries (de la Torre-Ubieta et al. 2018;  supplementary tables S1 and S7, Supplementary Material online). We identified that younger lncRNAs are significantly depleted from the cortical germinative zones but not from the cortical plate (Fig. 5a).

To further explore the cellular context in which lncRNAs from different MA groups are expressed, we reanalyzed public scRNA-seq data (Fan et al. 2018; Zhong et al. 2018), mapping the cortical lncRNAs to the cellular populations of the human developing cerebral cortex. All identified cell populations (Fig. 5b) DE at least one member of each MA group (Fig. 5c; supplementary table S7, Supplementary Material online), indicating widespread expression of all cortical lncRNAs. Of note, lncRNAs are enriched in mature excitatory neurons (Cajal–Retzius, synaptogenic glutamatergic, and mature subplate [SP] neurons), and several cortical cell populations preferentially express lncRNAs from certain MA groups. Thus, old lncRNAs (300 Mya) are enriched in all interneuron (GABAergic) cell populations (Inh. CGE, Inh. SST, Inh. MGE), oRGCs, and migrating glutamatergic neurons; instead, young lncRNAs (25 Mya, human-specific) are preferentially enriched in mature SP and synaptogenic glutamatergic neurons (Fig. 5c). In line with the depletion from germinative zones, cortical lncRNAs are depleted from cycling cell populations (apical and oRGCs, mGPC, intermediate progenitor cells [IPCs], microglia, oligodendrocytes, astrocytes, and oligodendrocyte progenitor cell), except for old lncRNAs that are enriched in oRGCs; at the same time, protein-coding genes are also enriched in those cell populations (Fig. 5c).

It has been shown that lncRNAs with low expression in bulk RNA-seq data are specifically expressed in a single-cell population in the developing cerebral cortex (Liu et al. 2016). Therefore, we assessed the specificity of cortical lncRNA expression in all gene categories. Of all gene categories considered, protein-coding genes are the most broadly expressed among cell clusters, as only 41% of protein-coding genes were specific to a single-cell population (Fig. 5d). Among lncRNA MA groups, old lncRNAs are found to be more broadly expressed among several cell populations (only 46% of lncRNAs from this MA group are specific to a single-cell cluster). In comparison, younger lncRNAs share a similarly high percentage of cell-type specificity (78% to 70%; Fig. 5d). Next, we assessed the cell-type enrichment of the cell-type–specific lncRNAs (Fig. 4e) and found that Cajal–Retzius cells, mature SP neurons, and synaptogenic glutamatergic neurons are the only cell types expressing significantly higher absolute numbers of cell-type–specific lncRNAs compared with other cells (Fig. 5e). Of the cell-type–specific lncRNAs, large numbers of mammalian-, primate-, and human-specific lncRNAs are enriched in Cajal–Retzius cells, while primate- (25 Mya) and human-specific lncRNAs are enriched in synaptogenic glutamatergic neurons; finally, mature SP neurons are enriched in human-specific lncRNAs (Fig. 5e).

### Cortical lncRNAs Are Regulated by a Set of Specific TFs

**Fig. 6.**
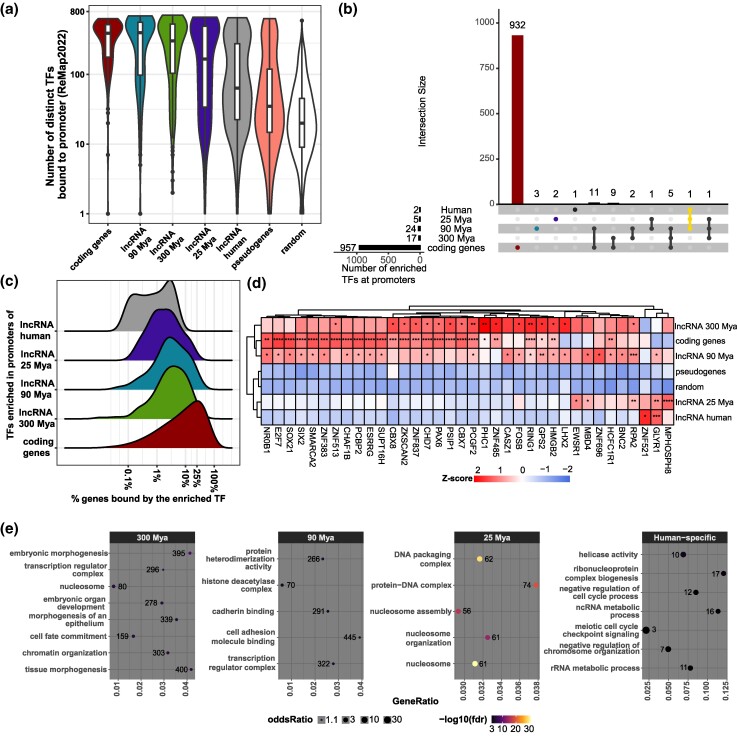
Regulatory landscape of cortical lncRNAs. a) Number of different TFs bound to the gene promoter region of genes in the gene category indicated at the *x* axis. Note the log scale used in the *y* axis. b) UpSet plot displaying the number of shared and specific TFs enriched in promoters of lncRNA MA groups and of protein-coding genes. c) Distribution of the frequency of all genes bound by an enriched TF in their promoter. TFs enriched in the promoters of genes from the different MA groups or enriched in the promoters of protein-coding genes were separately analyzed. d) Heatmap of the frequency of TFs bound in promoters of gene groups. Heatmap colors are proportional to the scaled frequency by gene group. e) GO enrichment of protein-coding genes having TFs enriched in the promoter of different lncRNA MA groups bound to their promoters. The number of TF-bound protein-coding genes identified in each GO category is given next to the circle; the significance of the enrichment is given by the color of the circle, according to the color scale at the bottom of the panel. Statistics: All statistics labels are FDR-corrected *P*-values of one-sided (greater) Fisher hypergeometric tests. *P ≤ 0.05; **P ≤ 10^−5^; ***P ≤ 10^−10^; ****P ≤ 10^−15^.

To better understand the molecular basis of the differential cellular distribution of cortical lncRNAs through evolution, we assessed their regulatory landscape using the Remap data (Hammal et al. 2022). We found that cortical lncRNAs present a higher number of TFs bound to their promoters than a set of random genomic regions and pseudogenes (Fig. 6a), speaking in favor that most of the identified lncRNAs are bona fide transcripts. Collectively, all cortical lncRNAs present a reduced diversity of TFs in their promoters compared with protein-coding genes. Nevertheless, older lncRNAs (300 and 90 Mya) show a higher variety of TFs bound to their promoters than younger lncRNAs (Fig. 6a), suggesting that older lncRNAs might be under a more complex regulation.

Next, we looked for the TFs that were statistically more frequent (enriched) in promoters of lncRNA MA groups. We found that most TFs were preferentially bound to promoters of protein-coding genes (Fig. 6b), few of them were more frequent in promoters of lncRNAs, and many of which were enriched in promoters of lncRNAs of the 300 and 90 Mya MA groups and shared occupancy at the promoters of protein-coding genes (Fig. 6b), in concordance with the higher diversity of TFs found in older lncRNA promoters. We then took the TFs that are enriched in the promoters of lncRNAs of each MA group and in the promoters of protein-coding genes (Fig. 6b) and looked for the distribution of the number of all genes that are bound by these TFs (Fig. 6c); we found that TFs enriched in the promoters of lncRNAs bind to 1% to 5% of all genes, whereas TFs that are more frequent in protein-coding promoters bind to 10% to 25% of all genes (Fig. 6c), indicating that lncRNA regulators, in general, are more specific. Furthermore, we found that TFs that were statistically more frequent in young lncRNAs (25 Mya and human-specific) were reduced in protein-coding gene promoters (Fig. 6d), implying that these TFs preferentially regulate lncRNAs. Additionally, we noticed that protein-coding genes regulated by TFs that were more frequent at the promoters of lncRNAs from each MA group are part of different molecular pathways (Fig. 6e and supplementary table S8, Supplementary Material online). TFs present in promoters of old lncRNAs (300 Mya) preferentially regulate protein-coding genes associated with embryonic morphogenesis (Fig. 6e), TFs present in promoters of mammalian-specific lncRNAs (90 Mya) preferentially regulate genes associated with histone deacetylase and cadherin binding (Fig. 6e), and TFs present in promoters of primate-specific lncRNAs (25 Mya) preferentially regulate histone genes (Fig. 6e). Finally, TFs present in promoters of human-specific lncRNAs preferentially control genes that negatively regulate the cell cycle process (Fig. 6e and supplementary table S8, Supplementary Material online).

Due to their specificity for regulating lncRNAs, we explored in more detail the set of TFs enriched in promoters of young lncRNAs. Most of the TFs enriched in promoters of primate-specific lncRNAs, namely, EWSR1, MDB4, and RPA2, regulated protein-coding genes involved in the nucleosome assembly process (supplementary fig. S7a and table S9, Supplementary Material online), and two of them, EWSR1 and RPA2, are expressed in IPCs (supplementary fig. S7b, Supplementary Material online). Interestingly, the GLYR1 TF is progressively enriched in mammalian-, primate-, and human-specific lncRNA promoters (Fig. 6d), is upregulated in synaptogenic glutamatergic neurons and Cajal–Retzius cells (supplementary fig. S7b, Supplementary Material online), and preferentially regulates genes involved in the negative regulation of the cell cycle (supplementary fig. S7a and table S9, Supplementary Material online), suggesting this TF as a driver of the postmitotic neural-specific expression of young cortical lncRNAs.

### Young lncRNAs Are Putative Sources of Molecular Innovation of Cortical Excitatory Neurons

**Fig. 7.**
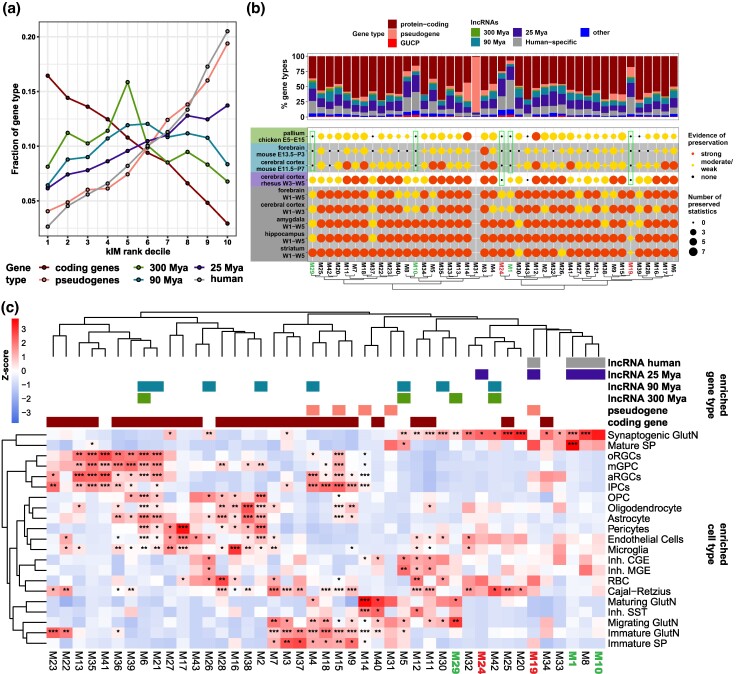
Young cortical lncRNAs contribute to novel molecular features of the developing cerebral cortex. a) Fraction of genes (*y* axis) from each gene category (colored as indicated at the bottom) present at the given intramodular connectivity (kIM) decile (*x* axis). In weighted gene coexpression modules, kIM indicates how well a gene is connected to other members of the gene modules. The first kIM corresponds to genes with the highest connectivity to other genes from the same gene coexpression module. b) Preservation analysis of 7 module statistics of cortical modules in coexpression networks from human, macaque, mouse, and chicken forebrain tissues (see Materials and Methods). **Strongly** preserved modules: all 7 module statistics were found preserved (Bonferroni-adjusted *P* < 0.05); **moderate/weak**: between 6 and 1 module statistics were found preserved; **none**: no module feature was found preserved. Fractions of gene types are displayed in the top panel. c) Intersection of cortical modules and scRNA-seq DE data, where the heatmap displays the odds ratio scaled by row. Colored blocks at the top indicate that a lncRNA MA group indicated at the top right is enriched in that gene module. Asterisk labels correspond to the statistical significance of the gene module enrichment at the given cortical cell types indicated on the right. Statistics: All statistics labels are FDR-corrected *P*-values of one-sided (greater) Fisher hypergeometric tests. *P ≤ 0.05; **P ≤ 10^−5^; ***P ≤ 10^−10^; ****P ≤ 10^−15^.

To understand how lncRNAs might have impacted the gene coexpression network of human cerebral cortex development, we employed weighted gene coexpression network analysis (WGCNA; Langfelder and Horvath 2008). After filtering library outliers, we used 187 bulk RNA-seq samples from the cerebral cortex from prenatal to early postconception, from which we identified 43 gene coexpression modules displaying different expression patterns throughout corticogenesis (supplementary fig. S8a and table S10, Supplementary Material online). Then, we assessed the centrality of the different lncRNA MA groups in these modules by comparing their intramodular connectivity (kIM) distribution. Protein-coding genes were significantly more central than all lncRNA MA groups (Fig. 7a). At the same time, cortical lncRNAs followed a gradient, where older lncRNAs were more central than younger lncRNAs, with human-specific lncRNAs following a distribution like pseudogenes (Fig. 7a).

Next, we assessed the possible role of cortical lncRNAs in gene network innovation using preservation network analyses, comparing seven module preservation statistics (see Materials and Methods; Ritchie et al. 2016) of the human gene coexpression network with the coexpression networks of other developing tissues of the human, macaque, mouse, and chicken forebrains (supplementary table S11, Supplementary Material online), and classifying them as strongly preserved or moderately/weakly preserved network modules as previously described (Ritchie et al. 2016; Zheng et al. 2021). We found that modules M19 and M24 were preserved only in humans and not in any other of the assessed species (Fig. 7b, black dots within green rectangles in chicken pallium, mouse forebrain and cortex, and rhesus cortex); also, modules M1, M10, and M29 were only moderately/weakly preserved with the rhesus macaque (Fig. 7b, yellow dots in rhesus cortex); therefore, we identified modules M19 and M24 as human specific, while modules M1, M10, and M29 as primate specific (Fig. 7b, green rectangles). Remarkably, most primate- and human-specific gene modules, namely, M1, M10, M19, and M24, were enriched in younger lncRNAs (Fig. 7b, top blue and gray color bars). When looking at protein-coding and lncRNA genes of the different MA groups that were expression matched to the 300 Mya lncRNAs (the group with the least number of lncRNAs), we observed that M1 remained enriched in younger human-specific lncRNAs and that the other three modules (M10, M19, and M24) remained with a high frequency of human-specific lncRNAs (supplementary fig. S8b, Supplementary Material online), indicating that the low level of expression of lncRNAs compared with protein-coding genes is not determining the pattern of enrichment.

When intersecting with the scRNA-seq DE data, it was possible to identify the cortical cell types enriched in these more divergent modules; in particular, excitatory neurons (synaptogenic glutamatergic and mature SP neurons) are highly enriched in primate- and human-specific coexpression modules (Fig. 7c), thus indicating that de novo expression of these young lncRNAs has predominantly occurred in the specialized excitatory neurons.

### Human-Specific lncRNAs Are Dysregulated in Neuropsychiatric Disorders

**Fig. 8.**
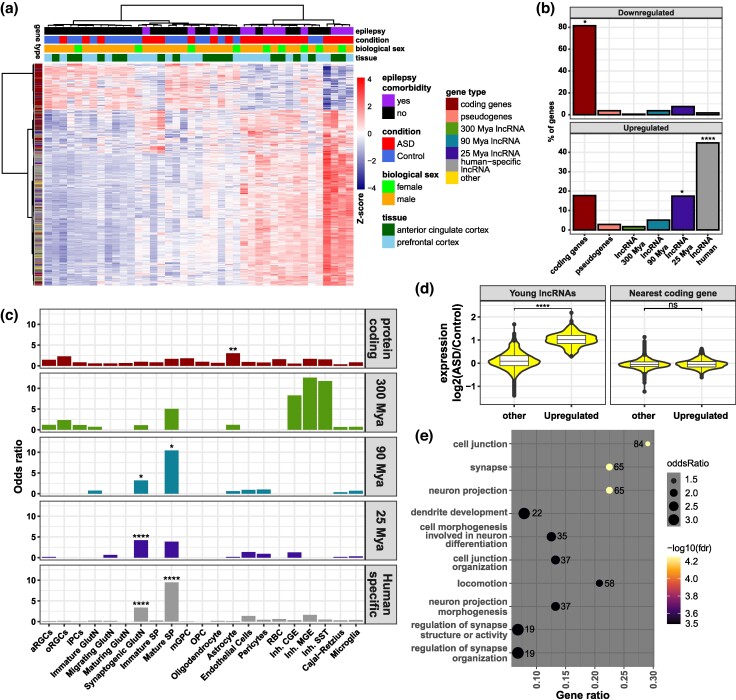
Young lncRNAs are dysregulated in neurodevelopmental disorders. a) Heatmap (unsupervised clustering) of DEGs in samples from individuals with ASDs, half of them with epilepsy comorbidity, compared with control individuals. The heatmap color indicates the *z*-score for each gene (line) calculated across all samples (columns) and is indicated by the color scale on the right. Sample characteristics are indicated by colors in the top panels and specified in the legend on the right. Gene types are shown in the far-left column, and their color legend is on the right. b) Percentage of DEGs, either downregulated (upper panel) or upregulated (lower panel) in ASD or ASD with epilepsy comorbidity relative to control samples, belonging to a gene category: protein-coding genes, pseudogenes, or one of the lncRNA MA groups. c) Enrichment of upregulated genes in specimens with ASD or ASD with epilepsy comorbidity expressed in the cell types of the developing cerebral cortex indicated in the *x*-axis, separated by gene category (each of the five panels). d) Log2-fold change expression differences of upregulated young lncRNAs (primate- and human-specific, left panel) in specimens with ASD or ASD with epilepsy comorbidity and other (non-DE) genes, and expression of their most proximal protein-coding genes (right panel). e) GO enrichment of most proximal protein-coding genes of young cortical lncRNAs upregulated in specimens with ASD or ASD with epilepsy comorbidity. The number of most proximal protein-coding genes identified in each GO category is given next to the circle; the significance of the enrichment is given by the color of the circle, according to the color scale at the right of the panel. Statistics: Labels represent FDR-corrected Fisher hypergeometric *P*-values. ns: *P* > 0.05; *P ≤ 0.05; **P ≤ 10^−5^; ***P ≤ 10^−10^; ****P ≤ 10^−15^.

Several studies have shown that neuropsychiatric disorders disrupt the homeostasis of postmitotic developing glutamatergic neurons (Parikshak et al. 2016; Li et al. 2018; Ziffra et al. 2021). As young lncRNAs are conspicuously expressed in this cell population and involved in its molecular diversification, we investigated whether cortical lncRNAs are dysregulated in neuropsychiatric disorders. Public bulk RNA-seq data from cortical tissue specimens from normal and autism spectrum disorder (ASD) patients, half of them with epilepsy comorbidity (Velmeshev et al. 2019), were subjected to DE analysis (Fig. 8a and supplementary table S12, Supplementary Material online). Remarkably, we identified that young cortical (primate- and human-specific) lncRNAs are highly dysregulated in ASD or ASD with epilepsy comorbidity (Fig. 8a and b). These upregulated young lncRNAs are preferentially expressed in synaptogenic glutamatergic and mature SP neurons (Fig. 8c). Despite the high dysregulation of young cortical lncRNAs, especially human-specific lncRNAs, we did not identify differences in the expression of the most proximal protein-coding genes (Fig. 8d). Nevertheless, these protein-coding genes proximal to upregulated young lncRNAs are involved in neurite development and synapse formation (Fig. 8e and supplementary table S13, Supplementary Material online), two disrupted functions in ASD or ASD with epilepsy comorbidity.

## Discussion

### Comprehensive Annotation of lncRNAs and Estimation of Their MA

Corticogenesis is a highly dynamic process that integrates many cells from different developmental regions; in particular, human corticogenesis is lengthy and comprises more diverse cells than other mammals (Molnár et al. 2019; Libé-Philippot and Vanderhaeghen 2021). Consequently, many lowly expressed lncRNAs or lncRNAs restricted to rare cell types of the developing cerebral cortex might not be annotated in public references such as Gencode (Frankish et al. 2019) or Ensembl (Yates et al. 2019), which is detrimental to studying the evolution of lncRNAs and limits our understanding of the transcriptional complexity of this tissue. A critical contribution of the present work was annotating a comprehensive complement of lncRNAs for humans, rhesus macaques, mice, and chickens. The large number of libraries used here, comprising the different periods of prenatal brain development in the four studied species (supplementary fig. S1, Supplementary Material online), and the stringent criteria used to classify annotated locus as coding or non-coding enabled us to identify new lncRNAs, pseudogenes, and protein-coding genes and to increase the number of reads mapped to the annotated transcriptomes compared with reference annotations (supplementary figs. S4i to l and S5, Supplementary Material online), representing a qualitative and quantitative improvement.

Estimating the evolutionary ages of the lncRNA repertory of humans is key to characterizing its evolution. Based on the concept of syntenic conservation of lncRNAs as an indicator of homology (Hezroni et al. 2015), we developed a computational method that required reciprocal genomic positional conservation to identify homologous lncRNAs between humans and the other three assessed species. By adding the syntenic conservation annotation of lncRNAs from previous studies (Hezroni et al. 2015; Sarropoulos et al. 2019; Bryzghalov et al. 2020), we classified all human lncRNAs annotated in our comprehensive transcriptome into different minimal evolutionary groups. We documented that these groups present a distinct distribution of TEs in lncRNAs, with younger lncRNAs having a progressively higher frequency of primate-specific Alu insertions (Fig. 3c and d). Also, we showed a depletion of younger lncRNAs in germinative zones and their increased expression in postmitotic neurons. This aligns with the reported high expression of species-specific lncRNAs at late prenatal stages (Sarropoulos et al. 2019), as well as stronger transcriptional processing and splicing efficiency in older lncRNAs, as previously reported (Hezroni et al. 2015; Ulitsky 2016). In summary, this indicates the good quality of MA estimation. The comprehensive transcriptome annotations and this evolutionary age annotation are now available and can be used in future studies of lncRNA evolution.

### Cortical lncRNAs Display Distinct Genomic and Transcriptional Features throughout Evolution That Suggest Different Evolutionary Histories and Functionalities

We found notorious differences in the RNA processing among MA groups. Older lncRNAs achieve higher expression than younger lncRNAs; especially old lncRNAs have as much expression as mRNAs (Fig. 2b). Additionally, older lncRNAs present features of higher splicing activity as they have shorter and more exons, larger introns, higher frequency of strong canonical splicing motifs, longer transcripts, and more isoforms (Fig. 2c to h), which indicates selection against early transcriptional termination. These differences in transcription activity and processing might result from the evolutional genesis requirements of lncRNAs, which are consistent with the proposed constructive neutral evolution regime (Palazzo and Koonin 2020). This evolution model postulates that de novo expression of lncRNAs by pervasive transcription of RNA pol II or TE transposition is first maintained by the selection of RNA transcription per se and then by additional modularity gain during evolution (Palazzo and Koonin 2020). In line with this, older lncRNAs also contain larger fractions of TEs in their gene body, which increases the probability of acquiring new *cis*-regulatory elements while producing larger transcripts, a feature associated with gaining functionality (Engreitz et al. 2016; Ulitsky 2016). Altogether, it is possible to suggest that older lncRNAs might have gained gene regulatory functionalities through their transcription processing or by the acquisition of *cis*-regulatory elements from TEs. That said, we understand that not necessarily all lncRNAs identified in the present study might have a measurable phenotype associated with them; nevertheless, it is more likely that older lncRNAs have gained one.

Furthermore, we found differences in the genomic distribution, transcriptional regulation, and expression patterns throughout the evolution of cortical lncRNAs. Older lncRNAs are proximal to developmental regulatory genes such as homeobox TFs and microRNAs (Fig. 4) and the promoters of old lncRNAs are bound by a higher diversity of TFs than younger lncRNAs; some of them also bind to the promoters of protein-coding genes involved in broad developmental processes (Fig. 6e). Moreover, old lncRNAs are not depleted from cortical germinative zones and have a wider expression in different cell types of the developing cerebral cortex than younger lncRNAs (Fig. 5a, c, and d). Especially older lncRNAs are highly expressed in oRGCs, whose population is expanded in human cortical germinative zones and in inhibitory GABAergic interneurons, a diverse and, at the same time, a transcriptionally conserved neural type of the cerebral cortex (Tosches and Laurent 2019; Yu et al. 2021).

A good example of an old cortical lncRNA identified in our work is *DLX6-AS1*, a conserved lncRNA with two functionally characterized isoforms that regulate the development of inhibitory GABAergic neurons by controlling the expression of nearby *DLX5/6* TFs (Cajigas et al. 2018). Another old lncRNA we identified expressed in the human developing cerebral cortex is *OIP5-AS1* (also known as *Cyrano*), which is expressed in zebrafish, mouse, and human brains (Kleaveland et al. 2018). *Cyrano* helps to preserve cell proliferation in embryonic stem cells (Ulitsky et al. 2011) and mediates the target-directed microRNA degradation of miR-7 helping to maintain proper neural activity (Kleaveland et al. 2018). Overall, our results suggest that old lncRNAs have evolved and some of them might likely act as regulators of conserved developmental programs, possibly by various previously described mechanisms, including serving as a source of or competing with microRNA or acting as *trans*- and *cis*-regulators (Statello et al. 2021).

In contrast, cortical lncRNAs that evolved after the divergence of amniotes are preferentially expressed near genes that regulate neurite development, are depleted from germinative zones, and are conspicuously expressed in excitatory neurons (Cajal–Retzius cells, SP cells, and glutamatergic neurons; Figs. 4a and 5a, c, and e, respectively). Moreover, the promoter regions of primate- and human-specific lncRNAs are bound by a few TFs that showed a higher frequency in lncRNA promoters compared with other gene types and coregulated protein-coding genes negatively associated with cell cycle progression, which might explain their increased cell-type selectivity for excitatory neurons and their depletion from germinative zones. Younger lncRNAs also have an increased frequency of *Alu* insertions, which has been linked with the formation of primate neurological networks (Larsen et al. 2018). Still, we also found that young cortical lncRNAs integrate human-specific gene coexpression modules enriched in mature glutamatergic neurons (Fig. 7b and c). These features suggest that some of these young cortical lncRNAs are tissue markers and might be relevant for the proper development and molecular diversification of mature excitatory neurons.

It is tempting to postulate that young unstable lncRNAs, which are enriched in the introns, or in the proximity of genes (Fig. 2a) involved in axon and dendrite development (Fig. 4a), may interact with template DNA of the RNA pol II to form R-loops to relax chromatin and allow prolonged transcription. In line with this idea, it was recently found that *Alu* sequences from eRNAs and upstream antisense lncRNAs help to mediate enhancer–promoter interactions (Liang et al. 2023). This could be an interesting mechanism of action for young lncRNAs, as the human cerebral cortex is characterized by a protracted development compared with other primates and mammals in general (Libé-Philippot and Vanderhaeghen 2021). Then, human excitatory neurons could use young cortical lncRNAs to sustain a relaxed chromatin state throughout the lengthy cerebral cortex development, a hypothesis that warrants future characterization.

### lncRNAs in Neuropsychiatric Disorders

The increased expression of young lncRNAs in cortical samples from patients with neural developmental disorders, such as ASD or ASD with epilepsy comorbidity, human-specific diseases characterized by defective synapsis, suggests that these young lncRNAs might play a role in the proper synaptogenesis of human neurons. Of note, we did not identify differences in the bulk expression levels of protein-coding genes nearest to lncRNAs affected in ASD or ASD with epilepsy comorbidity, which does not eliminate a possible function of these young lncRNAs in the proper mRNA transcription processing of their nearest genes, or differences at the single-cell-type level. Thus, we postulate that young cortical lncRNAs could have been used as a molecular source of evolution and might be involved in the dysfunction of human excitatory neurons, primarily in synaptogenesis.

## Conclusions

In conclusion, we systematically assessed the contribution of lncRNAs to human cerebral cortex evolution by annotating comprehensive catalogs of cortical lncRNAs in humans, rhesus macaques, mice, and chickens and by identifying the MA of human cortical lncRNAs. We showed that human cortical lncRNAs exhibit signatures that suggest they may play a role during functional specialization of the cortex throughout evolution.

Older lncRNAs are preferentially expressed near developmental regulatory genes such as homeobox TFs and microRNAs and are enriched in conserved cell types, such as oRGCs and inhibitory neurons, possibly working as a source of molecular innovation of conserved molecular programs. On the other hand, younger cortical lncRNAs are selectively expressed in postmitotic excitatory neurons, divergent neurons of the cerebral cortex, where they belong to primate- and human-specific coexpression networks and are dysregulated in neural developmental disorders such as ASD or ASD with epilepsy comorbidity, suggesting that they might be a source of molecular evolution and dysfunction of excitatory neurons.

## Materials and Methods

### Tissue Collection, RNA Extraction, and Sequencing

*Gallus gallus* fertilized eggs were purchased from a local provider and incubated at 38 °C and 50% humidity for 7 and 10 days. Embryos were collected and decapitated; brains were removed from the heads, and forebrains were further dissected. For developmental day 7 (E7), the whole pallium and subpallium were retrieved; for developmental day 10 (E10), the entire subpallium, the dorsolateral pallium, and the medial pallium were retrieved. Three brain sections from different embryos were pooled per working sample without considering biological sex. Brain sections were dissociated using pestles in 1-mL TRIzol and frozen at −80 °C until the day of RNA isolation.

For RNA isolation, 200 µL of chloroform was added per 1-mL TRIzol and centrifuged for 15 min and 16,000 × *g* at 4 °C; supernatants were transferred to new microtubes. One volume of 100% ethanol was added to each sample and then transferred to RNeasy Mini spin columns. After that, the RNeasy Microkit protocol was followed.

RNA samples were quantified using a Qubit2 Fluorometer (Thermo Fisher), and their integrity was measured using a Bioanalyzer 2100 (Agilent). The RNA integrity number of the samples ranged from 7.5 to 8.5, indicating good quality. Four biological replicates were prepared and sent for sequencing to BGI Genomics (Shenzhen, China) for each tissue developmental window.

### Bulk RNA-Seq Processing

Public libraries were retrieved from the SRA repository at GenBank (NCBI, USA) using *fasterq-dump* with the following parameter: “–-split-files”; the integrity of the data was checked using *vdb-validate*, and all files were identified as consistent. Mapped bam files for the rhesus macaques were retrieved from the Synapse repository (https://www.synapse.org/#!Synapse:syn17093056/tables/RhesusmRNA-seq) using the repository API for UNIX and then transformed into fastq files using the *bedtools bamtofastq* (Quinlan and Hall 2010) function. Raw fastq files generated in the present work and sequenced at BGI Genomics were retrieved from a dedicated AMAZON Web services account. Raw fastq files from all sources were then processed with *fastp* (Chen et al. 2018) to remove read adapters and to check read quality before and after trimming. Trimmed fastq files were mapped to the reference genome using *STAR* version 2.5.4b (Dobin et al. 2013) using the following parameters: “–-outReadsUnmapped Fastx–-chimSegmentMin 12–-chimJunctionOverhangMin 12–-alignSJDBoverhangMin 10–-alignMatesGapMax 100000–-alignIntronMax 100000–-chimSegmentReadGapMax 3–-alignSJstitchMismatchNmax 5 -1 5 5–-runThreadN 94–-outSAMstrandField intronMotif–-outFilterMultimapNmax 20–-outFilterType BySJout–-outFilterMismatchNoverReadLmax 0.04–-alignIntronMin 20–-outSAMtype BAM Unsorted”. The latest available genome assemblies and the following Gencode/Ensembl gene annotations were used as genome references: hg38 and Gencode v37, rheMac10 (Warren et al. 2020) and Ensembl 104, mm39 and Gencode m27 (https://www.ncbi.nlm.nih.gov/grc/mouse), and galGal6 (Bellott et al. 2017) and Ensembl 101.

The resulting unsorted BAM files were sorted using *samtools* (Li et al. 2009).

### Iso-Seq Long-Read Processing

Raw unmapped bam files from the SRA project PRJNA476474 were downloaded directly from the SRA repository using GNU wget for the rhesus macaque. The standard *Isoseq3* pipeline was used to obtain polished, high-quality fasta files for all processed samples. Additionally, for the chicken (*G. gallus*), fasta files from Iso-seq deposited at SRA were downloaded using *fasterq-dump*, as described above. Detailed information on all libraries used can be found in supplementary table S1, Supplementary Material online.

Long-read fasta files were mapped to the reference genomes using *Minimap2* (Li 2018) with the following parameters: “-ax splice -uf–-secondary = no -C5 -O6,24 -B4”. Output sam files were converted to bam files, sorted, and indexed using *samtools*. All output sam files from the same species were collapsed into a gtf file using the function *collapse_isoforms_by_sam.py* from *Cupcake* (https://github.com/Magdoll/cDNA_Cupcake/) with default parameters. Spurious transcripts were removed from the collapsed gtf file using the functions *sqanti3_qc.py and sqanti3_RulesFilter.py* from *Squnti3* (Li 2018) with default parameters.

### GTF Building

To generate consensus gene models from short reads, sorted bam files from bulk RNA-seq libraries were processed using *scallop* (Shao and Kingsford 2017) with the following parameters: “–-min_transcript_length_base 200–-min_mapping_quality 250–-min_splice_bundary_hits 1”. To choose the correct parameter for “–-library_type”, the type of library was assessed before running *scallop*; for the parameter “–-min_num_hits_in_bundle”, 10 was chosen if the library possessed less than 20 million uniquely mapped reads; otherwise, 20 was used. After generating gtf files for every sample, the monoexonic transcripts were removed from unstranded libraries. Additionally, the monoexonic transcripts were removed from rRNA-depleted libraries using *gffread* (Pertea and Pertea 2020) with the parameter “gffread in_gtf_file -F -U -T -o/out_put/file”.

GTF files from all samples of the same developmental window/brain region were merged into a consensus transcriptome using *taco* (Niknafs et al. 2017) before generating the final gtf files to avoid overrepresentation of libraries of a tissue/brain region group, which would bias the construction of the final transcript model, with the following parameters: “–-gtf-expr-attr RPKM–-filter-min-expr 0–-filter-min-length 200–-isoform-frac 0.01”. Consensus transcriptomes were merged into a final gtf file using *taco* with similar parameters. The output consensus file was filtered for readthrough, mapping errors, intron retention, and run-on polymerase transcripts using *gffcompare* (Pertea and Pertea 2020) with the species reference transcriptome (human, Gencode v37; rhesus macaque, Ensembl 104; mouse, Gencode m27; and chicken, Ensembl 101) as a model to generate the final consensus gtf file.

### Coding Potential Identification

Coding potential was assessed for all transcripts in the final gtf files using *CPAT3* (Wang et al. 2013), *FEELnc* (Wucher et al. 2017), and *CPC2* (Kang et al. 2017). For CPAT3 and CPC2, gtf files were transformed into fasta files, first generating intermediate bed files with *gffread*, then using *getfasta* from *samtools* with the following parameters: “-split -name –s”. The fasta files were used as input to generate coding potential values for each transcript. For FEELnc, the reference gtf files for each evaluated species were used to train the random forest model, running the tool with the following parameters: “-*n* 6000,6000–-learnorftype = 3–-testorftype = 3”. Tables with output coding potential scores can be found at https://doi.org/10.5281/zenodo.10038370.

### ORF Identification and Annotation

*Transdecoder* 5.5.0 (Douglas 2018) was used to identify *bona fide* ORFs; additional Pfam and UniProt matches were provided to improve the identification of ORFs. The *HMMER* 3.1b2 tool (Mistry et al. 2013) was used to identify Pfam matches with the following parameters: “hmmscan–-domtblout pfam.domtblout–-tblout file_name.tsv -E 1e-5”. To identify UniProt matches, *blastp* was used with the following parameters: “-max_target_seqs 1 -outfmt 6 -evalue 1e-5”. The final identification of ORFs was carried out using the function *TransDecoder. Predict* with the following parameters: “–-retain_pfam_hits pfam.domtblout–-retain_blastp_hits blastp.outfmt6”. Additionally, gff3 files were generated for each species containing the genomic coordinates of the ORFs, information that was added to the final consensus transcriptomes. Finally, eggNOG-mapper (Huerta-Cepas et al. 2017) matches were searched for all identified ORFs using the UNIX standalone tool *emapper* 2.0.1 with default parameters. Output annotations of identified ORFs were incorporated into the final comprehensive transcriptome that can be found at https://doi.org/10.5281/zenodo.10038370.

### TE Content Identification

Repetitive elements from each studied species were downloaded from the University of California Santa Cruz (UCSC) Genome Browser database (https://genome.ucsc.edu), keeping only the records from TE families SINE, LINE, LTR, DNA, Retroposon, and RC. TE tables were converted to bed files using custom *Rscripts* and sorted using UNIX *sort*.

To identify coding sequences (CDSs) with more than 50% of their gene body coming from a TE element, protein CDS bed files were intersected with TE bed files using the *bedtools intersect* function with the following parameters: “-s -wo” to ensure strand specificity of the intersection. The total sum of TE intersections was divided by the length of the CDS; CDSs with more than 50% of their gene body coming from a TE element were tagged as “transposable elements.”

To identify the TE class distribution in CDS, mRNA UTRs, pseudogenes, and lncRNAs, the bed file from each species' new transcriptome assembly was intersected with the TE bed file. TEs that intersected at least 10 bp with a gene were kept for further analysis. The percentage of the gene body containing TEs was calculated as the ratio of the total length of all TEs intersecting with a gene to the gene length.

### Annotation of lncRNAs into MA Groups

Two approaches were used to identify conserved lncRNAs between humans and the other studied species: *liftOver* alignment between the two species, followed by searching for positionally conserved lncRNAs, or whole-gene sequence mapping from one species to the genome of the other, followed by searching for positionally conserved lncRNAs. In both approaches, all isoforms from a gene were first merged into a metagene using the *bedtools merge* tool, generating new bed files of metagenes. Due to the repetitive nature of TEs, we removed them from the metagene models using *bedtools subtract*. FASTA files for metagene annotations without TEs were generated using *bedtools getfasta*.

In the *liftOver* alignment approach, a metagene bed file of a query species was lifted to the genome coordinates of the other target species using the standalone *liftOver* function from the UCSC Genome Browser and the UCSC-generated *over.chain* file of the pairwise alignment between the whole-genome sequences of the two species as input, with the following parameters: for comparisons involving chicken, “-minBlocks=0.01 -minMatch=0.01”; for comparisons involving the three mammalian species, “-minBlocks=0.01 -minMatch=0.10”. This considerable reduction of minimum threshold parameters was necessary to maximize the number of lncRNA genes transferred from one genome to the other.

In the whole-gene sequence mapping approach, the metagene fasta file of a query species was mapped to the genome of the other target species using *Minimap2* with the following parameters: “-ax splice -uf –secondary=no -C5 -O4,24 -A2 -B4 -G 100K”. The output sam files were converted to bam sorted files using *samtools* and then to bed files using *bedtools bamtobed*.

For both above approaches, *liftOver* alignment and whole-gene sequence mapping approaches, we repeated each analysis after inverting the query and target species; only reciprocally conserved lncRNAs identified in both analyses were further used.

Bed files containing mapped lncRNAs from one species to the other genome using both approaches were joined, prioritizing the transferred genes originated from the *liftOver* alignment approach when available. The final set of transferred gene coordinates was intersected with the coordinates of lncRNA genes from our new transcriptome assembly of the target species using the *bedtools intersect* tool, and lncRNAs with the same position and genome strand orientation having at least one neighboring one-to-one protein-coding ortholog, as determined with *liftOver,* in the same direction were classified as homologous lncRNAs.

TEs enormously contribute to the gene body of lncRNAs (Kapusta et al. 2013); removing TEs from the gene body of lncRNAs might have led to the misidentification of some homologous lncRNAs. Thus, the same approaches, *liftOver* alignment and whole-gene sequence mapping, were repeated a second time, now without removing the TE insertions from the lncRNA body. The homology annotation results from this second analysis were added to the final annotation when an older homologous lncRNA resulted from this analysis when compared with the previous one.

To complement our set of identified conserved lncRNAs, lncRNA syntenic conservation classification from other databases was added to the final annotation. The gtf files containing the lncRNA annotation information were retrieved (Hezroni et al. 2015; Sarropoulos et al. 2019), and all isoforms from the same gene in one gtf file were merged into a metagene using the *bedtools merge* tool. Metagene coordinates from public gtf files were compared with our metagene annotation using *gffcompare* with default parameters. For each lncRNA gene, the oldest homology classification was retained if our classification differed.

Our final transcriptome assemblies for the four species are available as gtf files at https://doi.org/10.5281/zenodo.10038370.

The final homology annotation of human cortical lncRNAs obtained in the present work is given in supplementary table S2, Supplementary Material online.

Using the annotation files of our new transcriptome assemblies for the four species (supplementary tables S2 and S3, Supplementary Material online), the number of new lncRNAs contributed by our analyses was counted; essentially the database of origin of each lncRNA is given by its ID_name, and the ID_names were used for counting the number of lncRNAs in our final assembly originating either from the public databases (Gencode or Ensembl) or from our new assembly.

In addition, a comparison of the lncRNAs in our new assemblies for the four species with the lncRNAs annotated in RNAcentral, a curated lncRNAs database, was performed using the *gffcompare* tool and the gff3 files with genome coordinates of RNAs annotated in the RNAcentral database release 24.0 of 2024 March 13 for the four animal species downloaded from http://ftp.ebi.ac.uk/pub/databases/RNAcentral/current_release/genome_coordinates/gff3/. The results of the comparisons are shown in supplementary tables S2 and S3, Supplementary Material online.

### Bulk RNA-Seq Quantification and Differential Expression Analysis

Gene expression was quantified by *FeatureCounts* from the *Rsubread* package (Liao et al. 2019) using the new assemblies for each assessed species as a reference with the following parameters: “allowMultiOverlap=T, countMultiMappingReads=F, juncCounts=T, nthreads=96”. The raw expression matrix was batch corrected for humans using ComBat-seq (Zhang et al. 2020), as in the original PsychEncode publication (Li et al. 2018). TPM values were calculated from raw expression matrices as previously shown by Zhao et al. (2021):


$$\mathrm{TPM}i=\;\frac{qi/li}{\sum_{j}\;(qj/lj)}*10^{6},$$

where TPM*i* is the TPM value of *gene i*, *qi* is the number of reads mapped in *gene i*, *li* is the length in kilobases of *gene i*, and $\sum_{j}(qj/lj)\;$ is the sum of counts/length ratios of all genes.

Only genes with a TPM value greater than 0.5 for all samples from developmental window/brain region pairs were kept for the subsequent analyses. Filtered TPM matrices were normalized using variance stabilization normalization (Huber et al. 2002).

To identify DE genes (DEGs), the R package edgeR (McCarthy et al. 2012) was used. Briefly, lowly expressed genes (less than 0.5 CPM in all samples of a variable) from raw count matrices were removed. Filtered matrices were used to identify DEGs using the quasi-likelihood test; the resulting *P*-values were false discovery rate (FDR) corrected, and all genes with an FDR less than 0.05 were identified as DEGs.

### Single-Cell RNA-Seq Processing

Fastq files were retrieved from SRA using fasterq-dump as described above but with the following parameters: “fasterq-dump -S -O/output/dir -e 94 --include-technical”. Fastq files were then mapped to the reference genome using *STARsolo* (Kaminow et al. 2021) version 2.7.9a with the following parameters: “--soloType CB_UMI_Simple --soloCBwhitelist/barcodes/dir --soloBarcodeMate 0 --soloBarcodeReadLength 0 --soloCBstart 1 --soloCBlen 8 --soloUMIstart 9 --soloUMIlen 8 --readFilesCommand zcat --runThreadN 94 --soloStrand Forward --clipAdapterType CellRanger4 --readFilesIn READ1.fq READ2.fq --soloCellFilter None --soloFeatures Gene Velocyto GeneFull --soloMultiMappers PropUnique”. Raw, sparse matrices from all samples were loaded into R (R: A Language and Environment for Statistical Computing [Internet] 2018) and merged using *Seurat* (Hafemeister and Satija 2019). Cells with fewer than 10 thousand and more than 1 million UMIs, fewer than a thousand detected genes, and more than 5% of all counts mapped to mitochondrial genes were removed from further analysis. Raw, sparse expression matrix was normalized using the sctransform method (Hafemeister and Satija 2019) while regressing by the percentage of expressed mitochondrial genes, cellular-cycle score, number of UMIs, and the number of identified genes using the following parameters: “method=“glmGamPoi,” vst.flavor=“v2”, variable.features.n=5000, vars.to.regress=c(“percent.mt”, “CC.Difference”, “nCount_RNA”, “nFeature_RNA”)”.

Single-cell clusters were identified using the Seurat function FindNeighbors considering the first fifty dimensions and the function FindClusters with resolution *2.5*. Markers for all clusters were identified using the functions FindAllMarkers with the following parameters: ““assay = “SCT,” test.use = “wilcox”, only.pos = T, logfc.threshold = 0.25, min.pct = .25, return.thresh = 0.05, densify = T”. Known cell population gene markers were used to annotate the identified clusters to different cortical cell types.

### Identification of Expression-Matched Genes

The R package “optmatch” (Hansen and Klopfer 2006) was used to identify the set of expression-matched genes among the evaluated gene categories, utilizing the mean–variance stabilized expression of all samples from the same gene as input.

### Identification of Genes Closest to lncRNAs

The *bedtools* function *closest* was used to identify the most proximal genes with the following parameters: “-d,” for which a metagene bed file for protein-coding genes and small RNAs was built for each species. Only genes located in the genome approximately 100 kb of lncRNAs were kept for the following analysis. GO analysis of the closest protein-coding genes was undertaken using the R package clusterProfiler (Yu et al. 2012; Wu et al. 2021) and using as input the list of nearest protein-coding genes to a MA category and the list of all closest protein-coding genes as background.

### Preservation Analysis of Module Statistics in Coexpression Networks

Coexpression network inference and module detection were performed on a per-tissue and a per-species basis with expression data from libraries specified in supplementary tables S1 and S11, Supplementary Material online, using WGCNA v.1.70.3 with the default parameters (Langfelder and Horvath 2008). Seven module preservation statistics were used to quantify whether the relationships and correlation structure between nodes composing each module were replicated or preserved when measured in a different data set (Ritchie et al. 2016). According to Ritchie et al. (2016) and Zheng et al. (2021), we defined strong evidence for a module's preservation in another tissue or another species if all seven test statistics achieved Bonferroni-adjusted *P* < 0.05, moderate/weak evidence if one or more, but not all, test statistics had Bonferroni-adjusted *P* < 0.05, and no evidence if no test statistics had Bonferroni-adjusted *P* < 0.05.

### TF Assay

Promoters from the most extensive transcript with the highest number of exons were retrieved from a set of expression-matched genes from all assessed gene categories. An equal number of random genomic regions were also generated using *bedtools shuffle* as a null group. Nonredundant remap 2022 data (Hammal et al. 2022) were retrieved and intersected with the set of working promoters using *bedtools intersect*. The absolute number of different proteins bound to each gene category and random genomic regions were compared using the Wilcoxon test. To identify significantly enriched TFs more frequently present in the promoter of a gene category, a one-sided Fisher hypergeometric test was performed between the set of random sequences and the promoters from the gene category. *P*-values were FDR corrected, and all TFs with an FDR less than 0.05 were identified as enriched in that gene category. To test the function of the TFs enriched in each gene category, all genes with the TF bound to their promoter were retrieved and used for GO analysis, using all genes with at least one TF enriched in their promoter as background.

### Statistical Analysis

All statistical plots and tests were obtained using the statistical package R version 4.1.0 (R: A Language and Environment for Statistical Computing [Internet] 2018).

## Supplementary Material

msae123_Supplementary_Data

## Data Availability

Raw chicken RNA-seq data sequenced for this project were deposited at the NCBI SRA repository under accession number PRJNA1002381. The list of public library accession numbers from different repositories (Synapse and SRA) used in the present work can be found in supplementary table S1, Supplementary Material online. The new cortical lncRNA transcriptome annotations, the identified minimal evolutionary age of human cortical lncRNAs, and their matching homologous lncRNAs in the other studied species are provided in supplementary table S2, Supplementary Material online. GTF files with the transcriptome annotations for all species used in the present project can be found at https://doi.org/10.5281/zenodo.10038370.
