# Supplementary material for: The Human Developing Cerebral Cortex Is Characterized by an Elevated De Novo Expression of Long Noncoding RNAs in Excitatory Neurons
Source: Mol Biol Evol. 2024 Jun 24;41(7):msae123. doi: 10.1093/molbev/msae123 (PMC11221658; doi:10.1093/molbev/msae123)
Supplement: msae123_Supplementary_Data [file msae123_supplementary_data.zip › 2024_05_10 Supplementary Figs S1 to S8.pdf]

Supplementary Figures S1 to S8

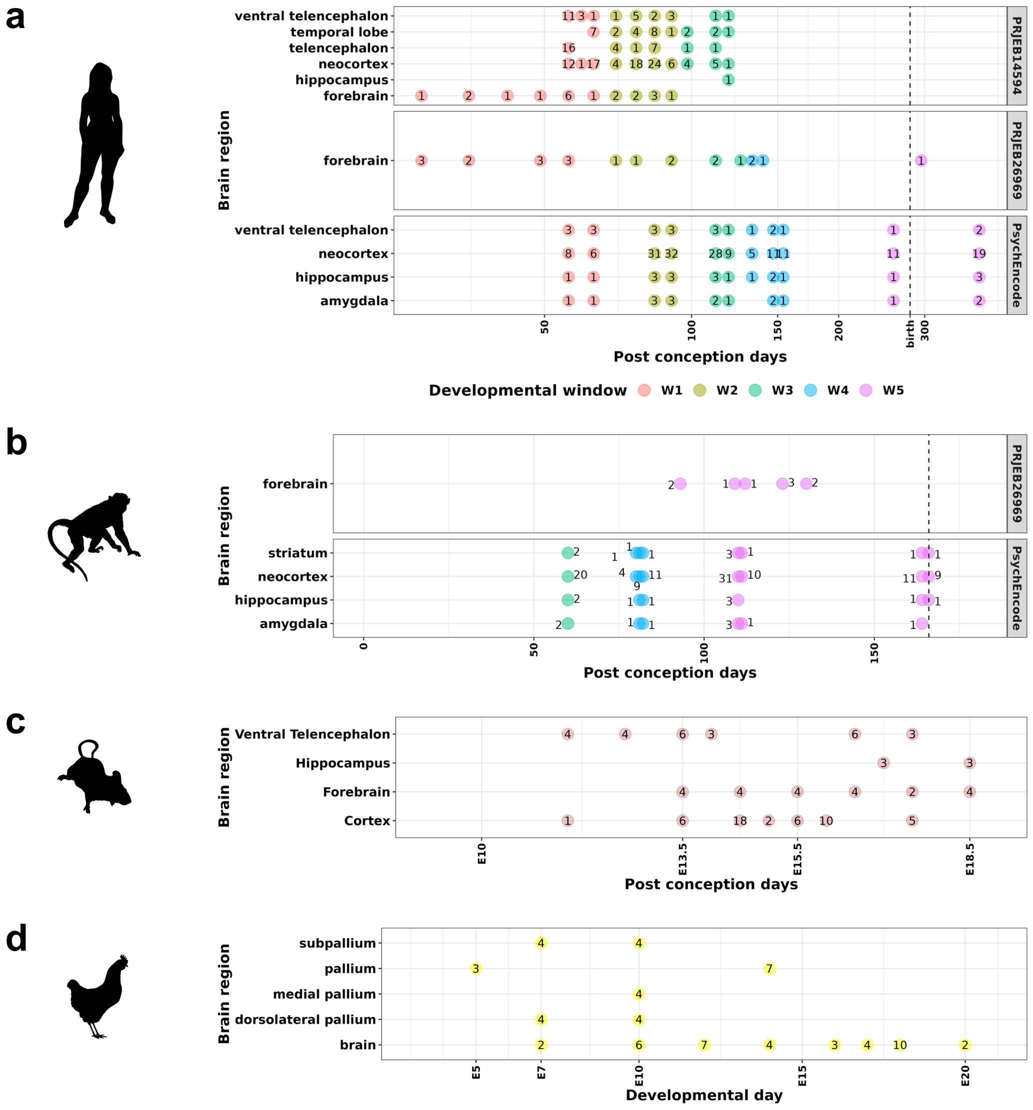

Fig. S1. Number of samples used for annotating new transcriptome assemblies. (a) Number of human samples (indicated inside the circles) used for building the transcriptome assembly of humans, grouped by the developmental time, tissue of origin, and the public data source (indicated at right), and colored by the PsychEncode developmental window. Developmental window W1, post-conception weeks 8 and 9; W2, post-conception weeks 12 and 13; W3, post-conception weeks 16 and 17; W4, post-conception weeks 19, 21 and 22; W5, post-conception week 37 and post-natal day 100. **(b)** Like in **a**, but for rhesus macaques. The PsychEncode developmental windows of macaques represent different developmental days but reflect similar developmental stages to humans. (**c)** Like in **a**, but in mice; there is no identification of matched developmental stages to humans, and samples span the cortical proliferative, early, and late neurogenesis stages. **(d)** Like in **a**, but in chickens; there is no identification of matched developmental stages to humans. Samples span the pallial proliferative, early, and late neurogenesis and gliogenic stages.

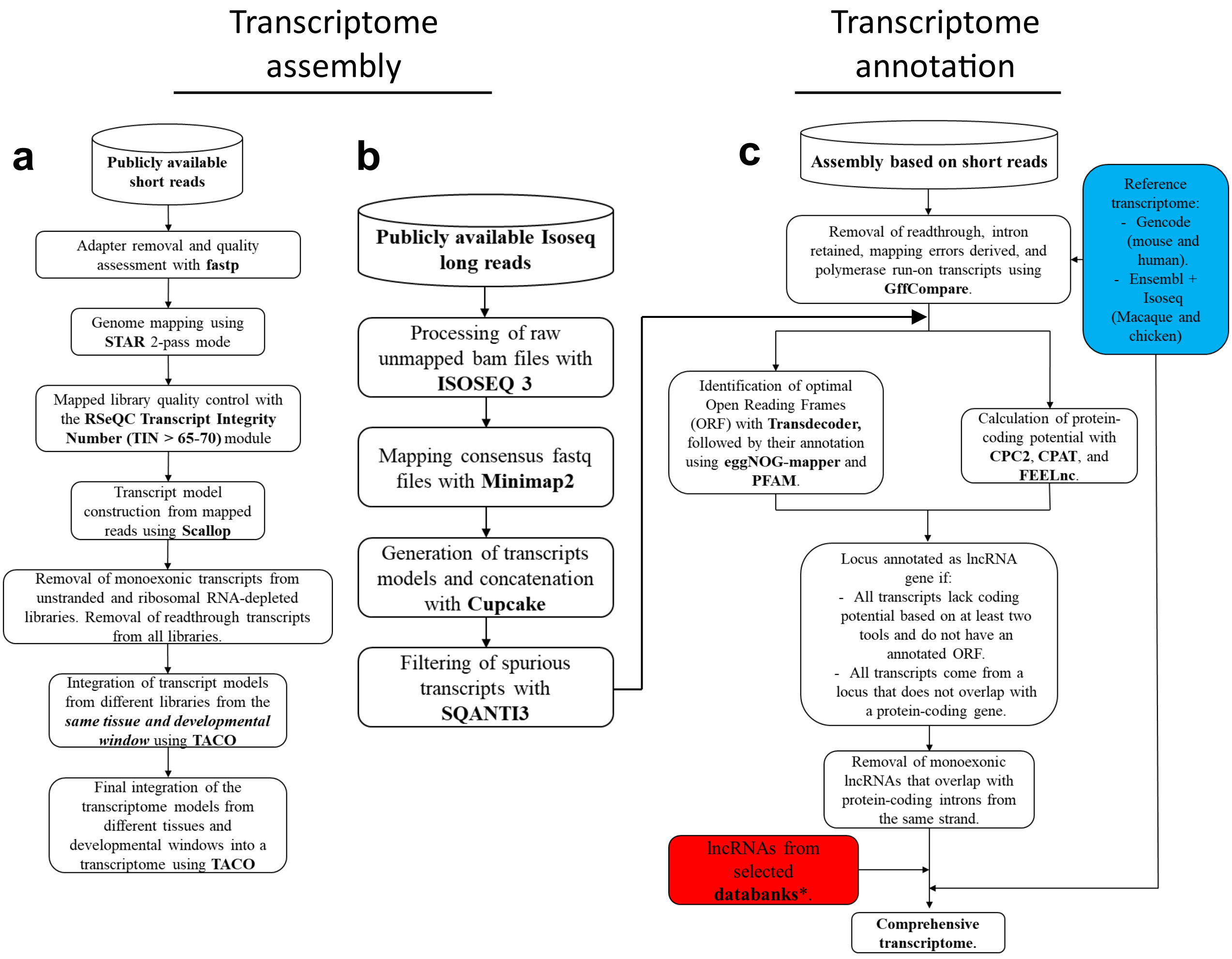

Fig. S3. Flowcharts depicting pipelines used for identifying and annotating comprehensive transcriptomes. (a) Bioinformatic pipeline developed to assemble new transcriptomes based on short reads. The pipeline uses *STAR* to map short reads, *Scallop* to build transcriptional models for each library, and *TACO* to generate consensus transcriptomes based on a set of transcriptional models. **(b)** Bioinformatic pipeline used to assemble new transcriptome models based on Iso-seq long reads. **(c)** Raw assembled transcriptomes built using short and long-read pipelines underwent extensive filters, first to remove spurious transcripts and to identify lncRNA genes and separate them from protein-coding isoforms. Additionally, transcripts from other public databases and the lncRNA set of reference transcriptomes were added to the final comprehensive transcriptome.

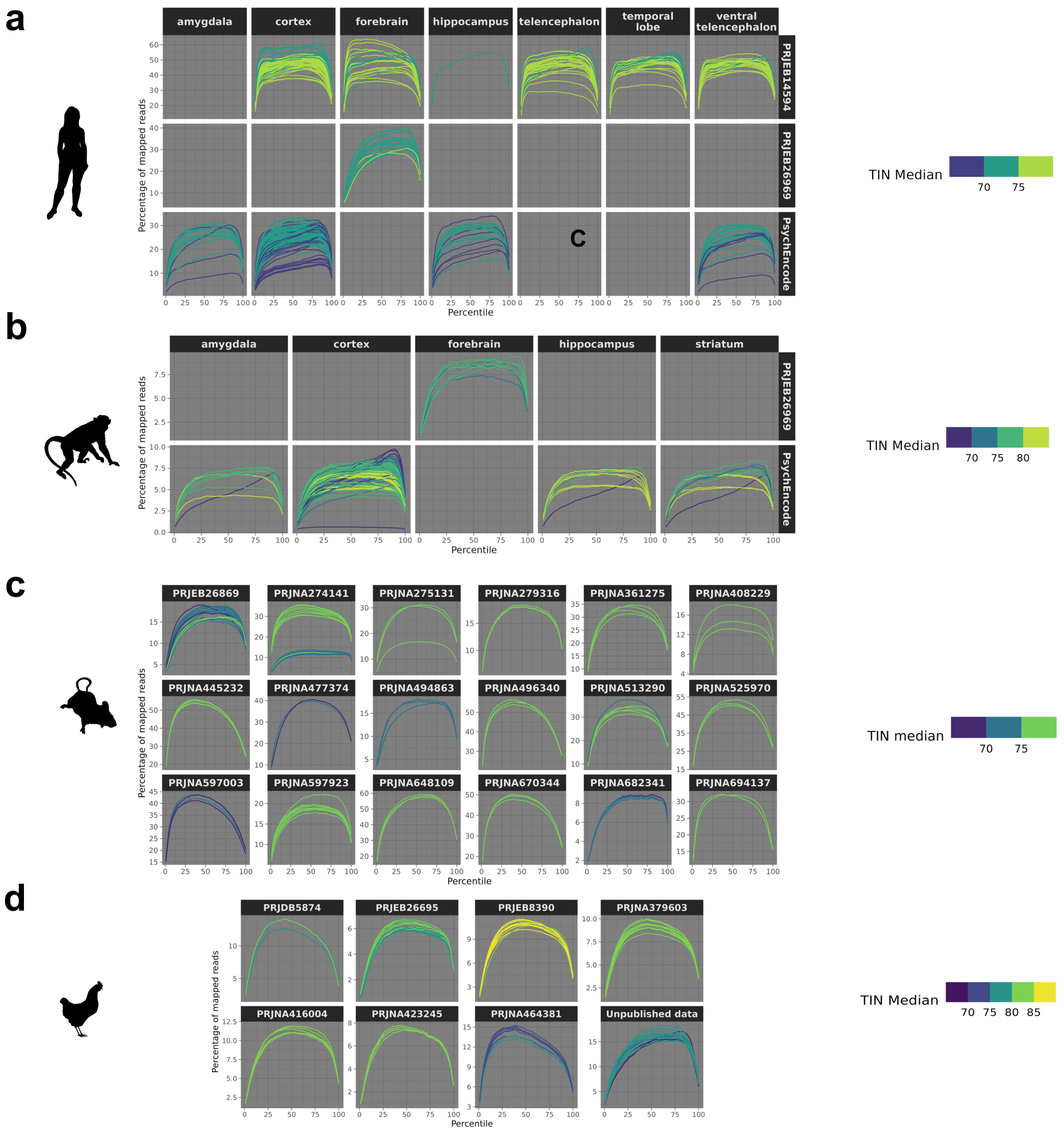

Fig. S2. Mapping quality of samples used to generate new transcriptome assemblies. **(a)** Averaged distribution of short read percentage along all transcript bodies in each library and colored depending on the Transcript Index Number (TIN) median score. Human library samples were separated by the public project and the tissues of origin. **(b)** Like in **a** but in the rhesus macaques. **(c)** Like in **a** but in mice, and libraries were separated only by the origin of public data. **(d)** Like in **a** but in chickens, and libraries were separated only by the origin of public data.

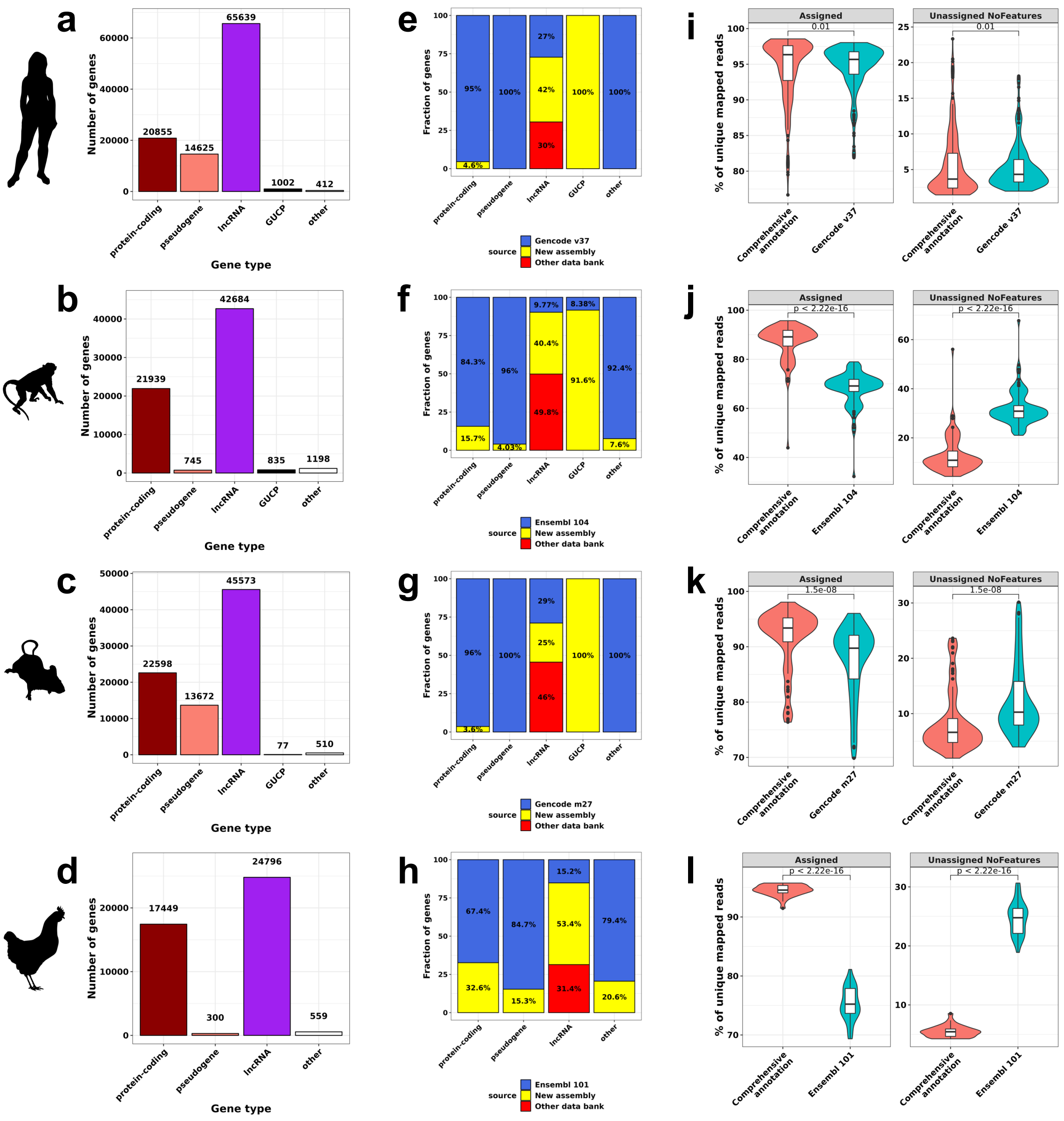

Fig. S4. New comprehensive transcriptome assemblies improve the annotation of lncRNAs. (a–d) Distribution of gene types in the new comprehensive transcriptomes annotated in the present work for humans, macaques, mice, and chickens, respectively. GUCP, genes of unknown coding potential. **(e–h)** Percentage of genes from different sources across the different gene types for humans, macaques, mice, and chickens, respectively. **(i–l)** Percentage of uniquely mapped reads, using as reference the present comprehensive annotation (red violin plots) or the Gencode and Ensembl public annotations (green violin plots), which mapped to an annotated region (Assigned, left panel) or to an unannotated region of the genome (Unassigned NoFeatures, right panel) for humans, macaques, mice, and chickens, respectively. **Statistics:** All statistics are one-sided (greater) Wilcoxon tests. ns: p greater than 0.05, *: p equal to or less than 0.05, **: p equal to or less than 0.01, ***: p equal to or less than 0.001, ****: p equal to or less than 0.0001.

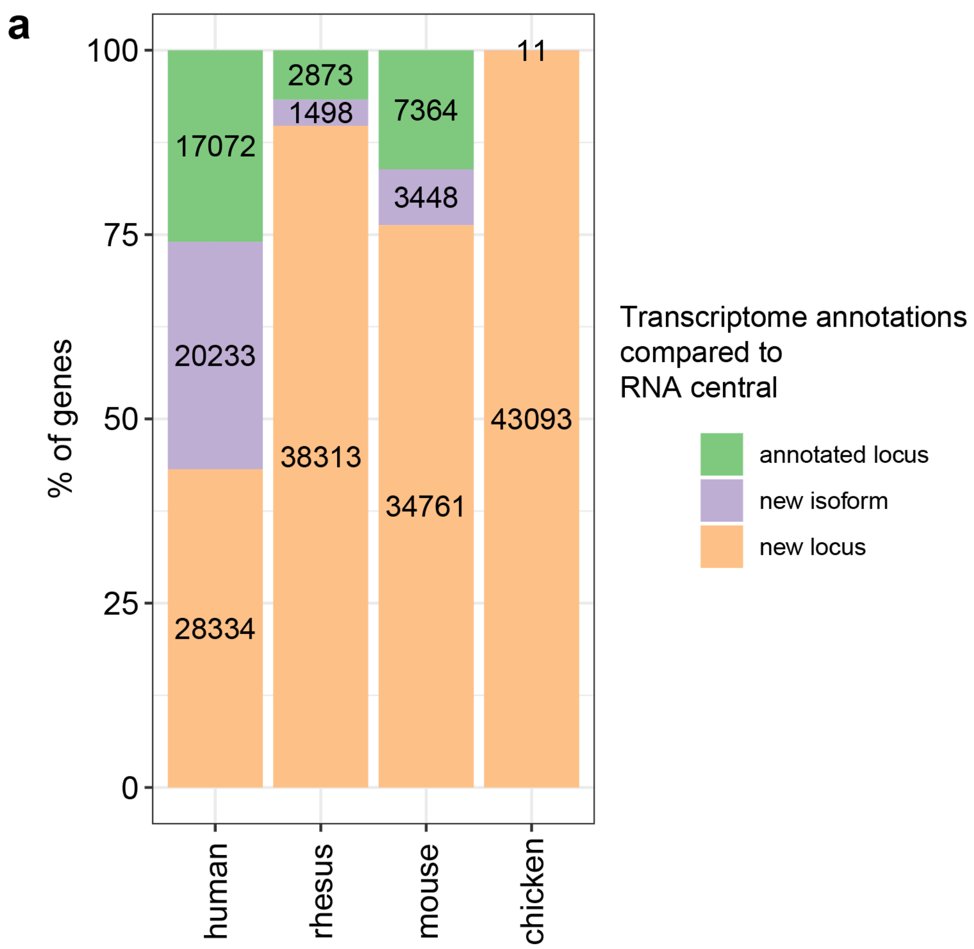

Fig. S5. Comparison of lncRNAs in our assemblies to lncRNAs in the RNAcentral database. Our transcriptome assemblies for each of the four species were compared with the *gffcompare* tool to the transcriptomes of the same species in the RNAcentral database. The percentage of our lncRNAs already present in RNAcentral are indicated as “annotated locus” (green); the percentage of our lncRNAs mapped as a new isoform in an RNAcentral genomic locus of another lncRNA are indicated as “new isoform” (violet); the percentage of our lncRNAs mapped to an RNAcentral genomic locus with no other lncRNA are indicated as “new locus” (orange).

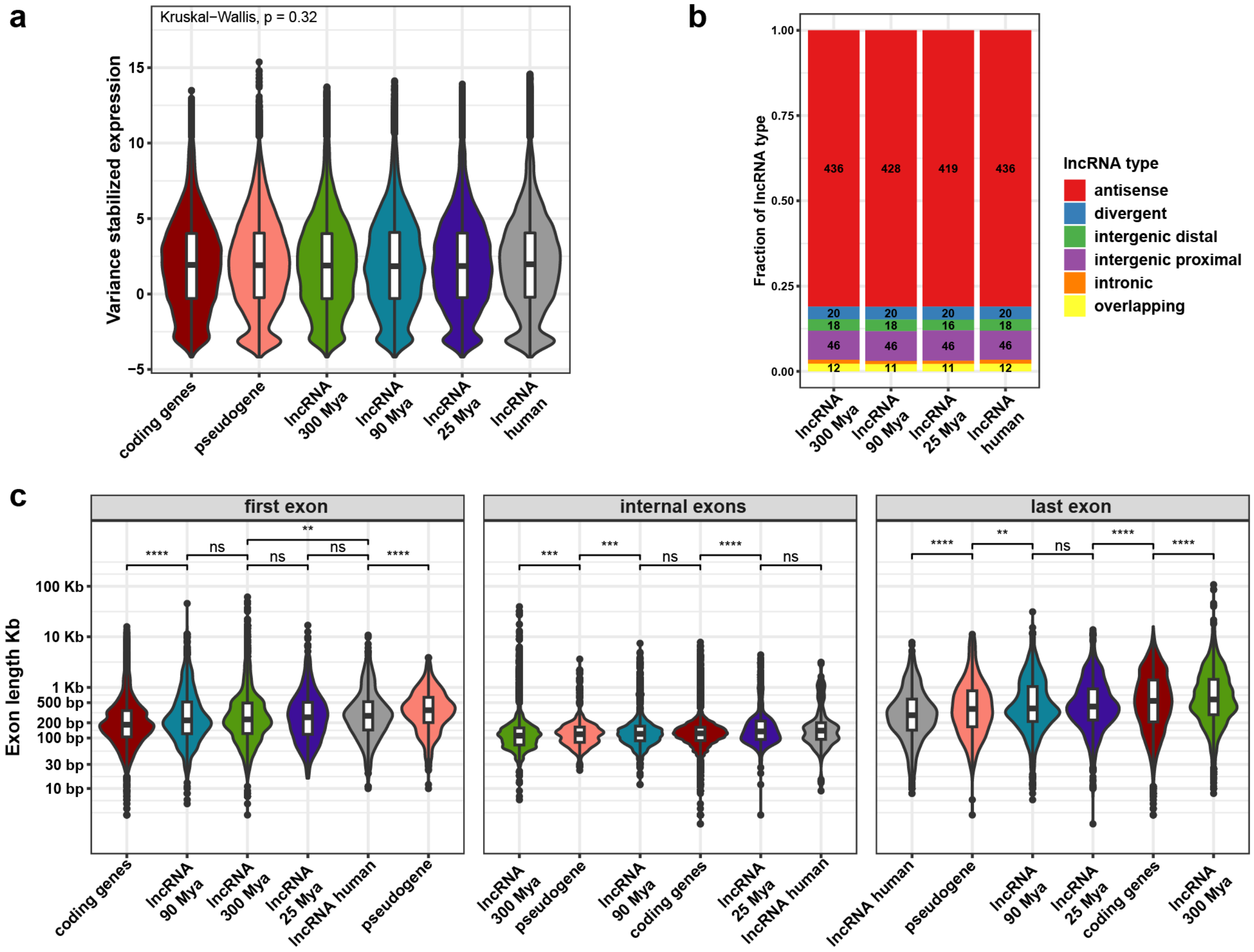

Fig. S6. Exon lengths of first, internal, and last exons among protein-coding genes, pseudogenes, and lncRNA MA groups. (a) Violin plot of the expression values of the expression-matched genes selected in each gene category indicated in the x-axis. **(b)** distribution of lncRNA types among the expression-matched genes selected in each lncRNA minimal-evolutionary-age (MA) group. **(c)** exon length distribution among all protein-coding genes, pseudogenes, and lncRNA MA groups present in our human transcriptome assembly, separated by first exon (left panel), internal exons (middle panel) and last exon (right panel). Note that the gene groups are ordered in each panel by the increasing median length from left to right. Statistics: All statistics are one-sided (greater) Wilcoxon tests. ns: p greater than 0.05, *: p equal to or less than 0.05, **: p equal to or less than 0.01, ***: p equal to or less than 0.001, ****: p equal to or less than 0.0001.

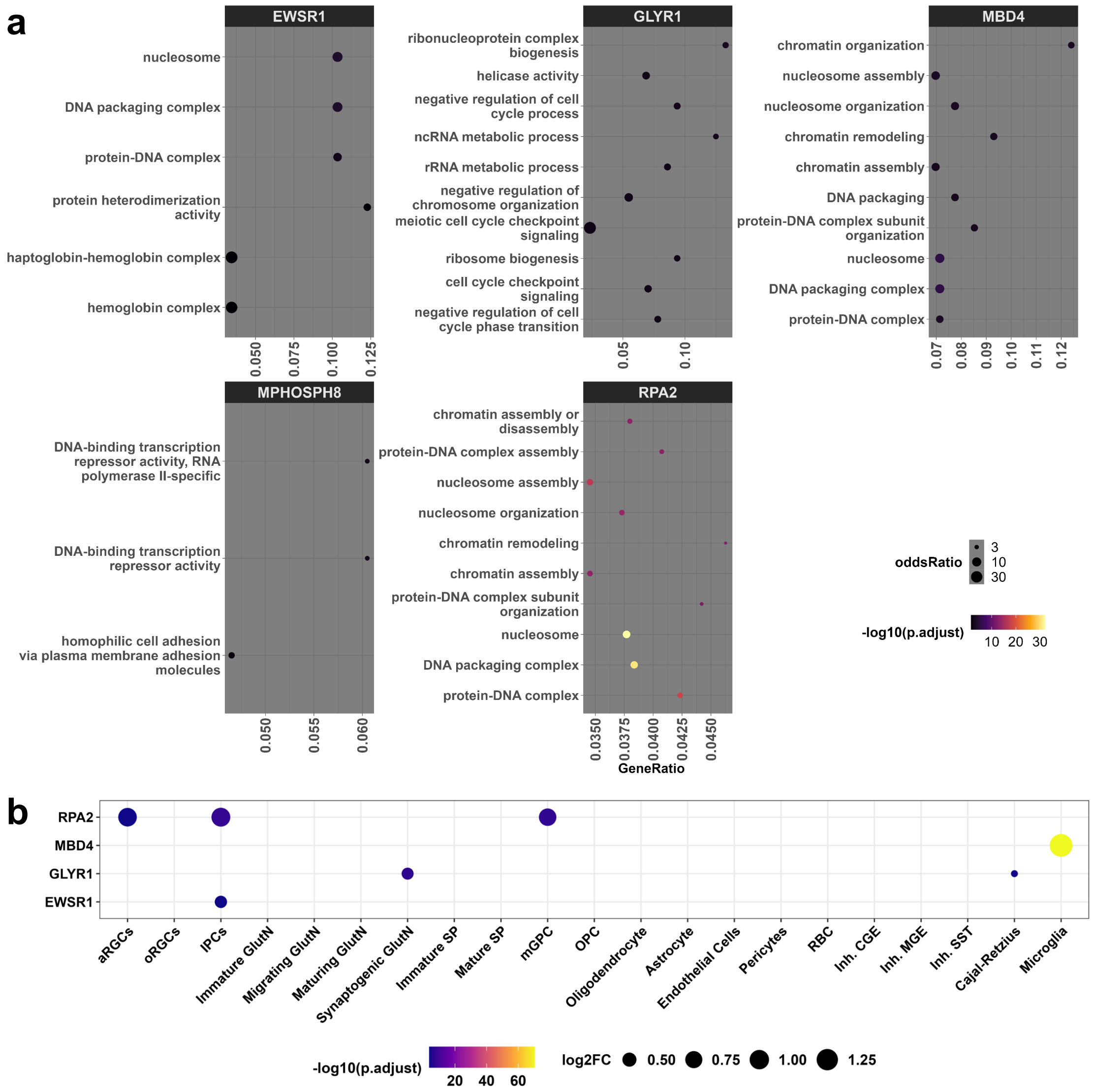

Fig. S7. Functional features of transcription factors enriched in the promoter of young cortical lncRNAs. (**a**) Gene ontology enrichment of genes regulated by the transcription factor (TF) indicated at the top of each panel, which is enriched in promoters of primate and Human-specific lncRNAs. (**b**) Dotplot showing the tissues of expression enrichment of each TF seen in **a**.

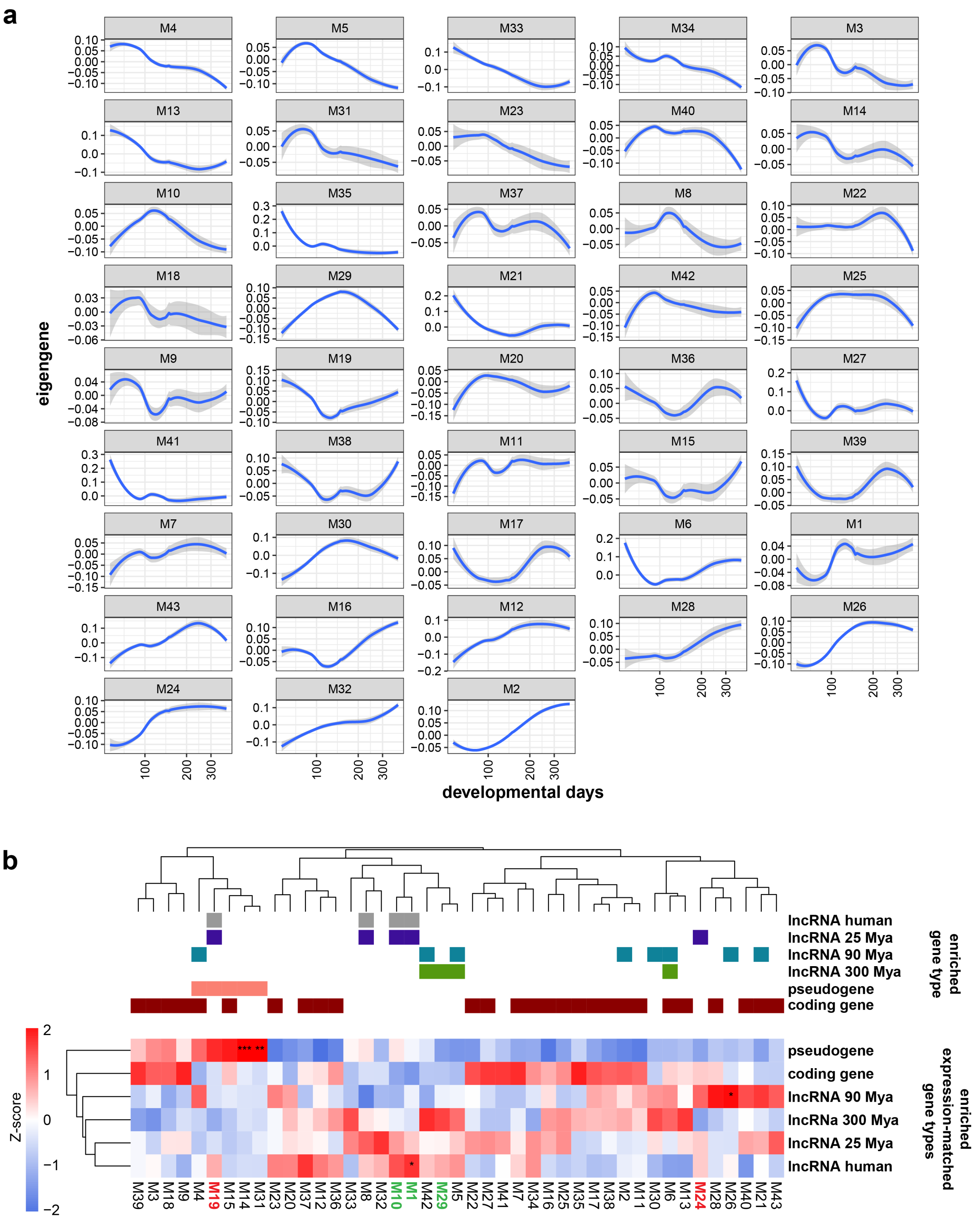

Fig. S8. WGCNA lncRNA trajectories along developmental days and lncRNA types enriched in each module. Expression-matched set of genes was selected (see Methods, Supplementary Fig. 6a and Supplementary Table 2). **(a)** Average eigengene value of each of the identified 43 co-expression modules along the human cerebral cortex development. **(b)** Heatmap displaying enriched gene type in each of the 43 gene co-expression modules.

Top: solid blocks represent enriched gene types based on all expressed genes. Bottom: display the Z-score normalized frequency of expression-matched genes across the modules within each group, and the asterisk indicate FDR-significant enrichment of a gene type within a module. Statistics: All statistics are one-sided (greater) Wilcoxon tests. *: p equal to or less than 0.05, **: p equal to or less than 0.01, ***: p equal to or less than 0.001.
